# Extracellular vesicles as mediators and markers of acute organ injury: current concepts

**DOI:** 10.1007/s00068-021-01607-1

**Published:** 2021-02-03

**Authors:** Birte Weber, Niklas Franz, Ingo Marzi, Dirk Henrich, Liudmila Leppik

**Affiliations:** Department of Trauma-, Hand- and Reconstructive Surgery, University Hospital Frankfurt, Goethe-University, Frankfurt am Main, Germany

**Keywords:** Extracellular vesicles, Exosomes, Trauma, Organ injury, Systemic inflammation, MicroRNAs

## Abstract

Due to the continued high incidence and mortality rate worldwide, there is a need to develop new strategies for the quick, precise, and valuable recognition of presenting injury pattern in traumatized and poly-traumatized patients. Extracellular vesicles (EVs) have been shown to facilitate intercellular communication processes between cells in close proximity as well as distant cells in healthy and disease organisms. miRNAs and proteins transferred by EVs play biological roles in maintaining normal organ structure and function under physiological conditions. In pathological conditions, EVs change the miRNAs and protein cargo composition, mediating or suppressing the injury consequences. Therefore, incorporating EVs with their unique protein and miRNAs signature into the list of promising new biomarkers is a logical next step. In this review, we discuss the general characteristics and technical aspects of EVs isolation and characterization. We discuss results of recent in vitro, in vivo, and patients study describing the role of EVs in different inflammatory diseases and traumatic organ injuries. miRNAs and protein signature of EVs found in patients with acute organ injury are also debated.

## Introduction

Severe trauma is one of the disorders with the greatest healthcare and economic impact in society today [1]. Worldwide, it is the leading cause of mortality in young adults, and involves the highest incidence of potential life years lost. In traumatized and poly-traumatized patients, quick, precise, and valuable recognition of presenting injury pattern is of outmost need for proper patient management as delayed diagnosis may cause secondary complications and exaggerate mortality and morbidity. Recently, intensive efforts have been made to identify indicators that are associated with the pathological processes of the disease and organ injury [2]. The heterogeneity of the injury cases makes it difficult to accurately assess the level of trauma, predict the clinical outcome, and optimize the therapy for individual patients; therefore ,the search of certain specific and sensitive biomarkers, which will help to overcome these difficulties is continuing. Exosomes (Exos), one subclass of EVs, which was described long time ago, and which role was re-considered recently, now appeared to be a promising biomarker candidate for the broad range of diseases. Despite the growing number of evidences confirming the role of Exos in physiological intercellular communication and their potential as biomarkers in some diseases and cancer entities [3], less is known about their role as mediators and markers of acute organ injury in the traumatized patients. In the following sections, we aim to provide an overview of existing literature with the focus on the role of Exo/EVs during acute organ injury. We focus on inflammatory diseases including sepsis and systemic inflammatory response syndrome (SIRS), traumatic brain injury, acute cardiac damage, acute respiratory distress syndrome (ARDS), and acute liver and kidney injury. Next to the individual organ damage, we also review studies focused on multiple trauma and Exos.

## Extracellular vesicles

**Fig. 1:**
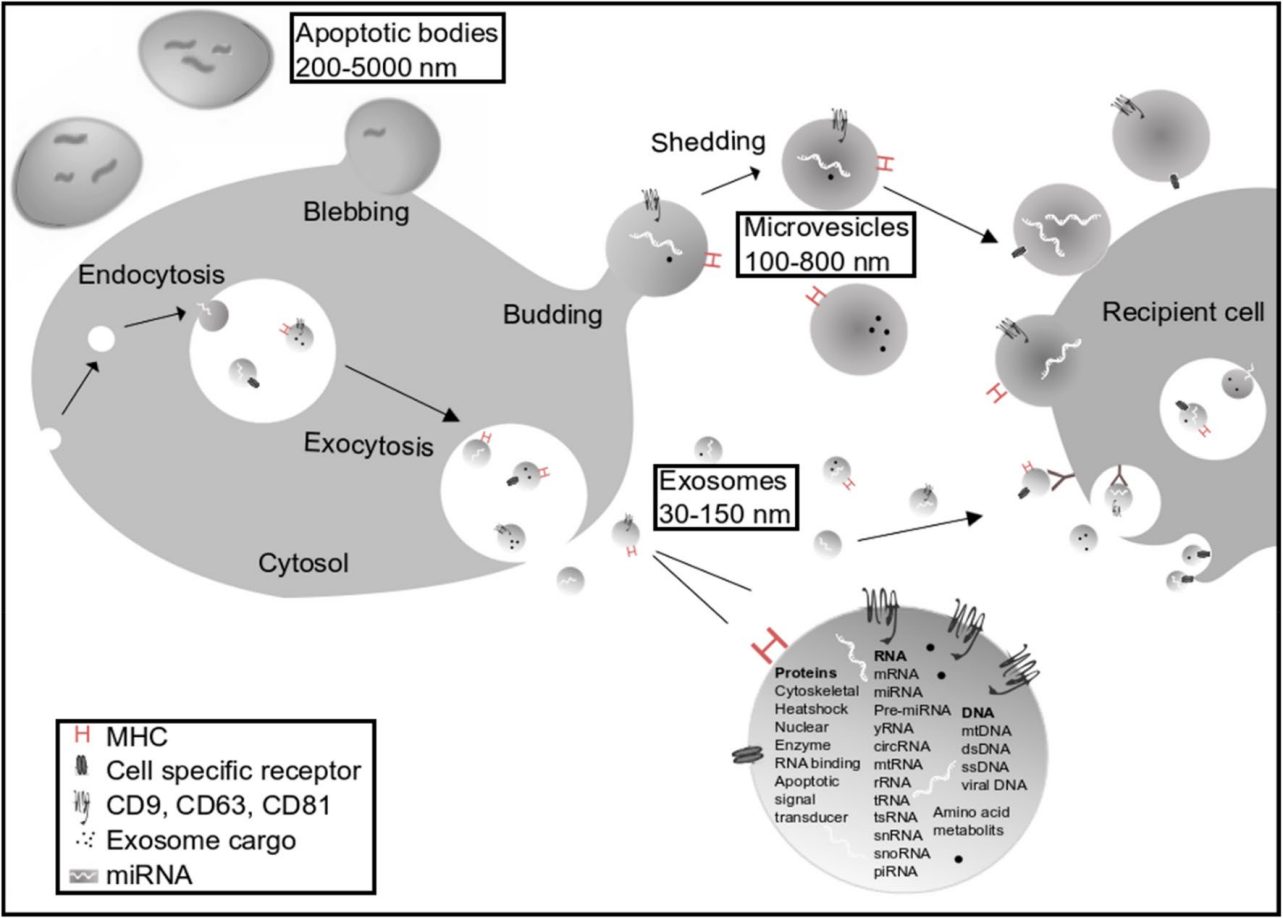
Extracellular vesicles (EVs): exosomes (Exos), microvesicles (MVs), and apoptotic bodies (ApoEVs). Schematic drawing of biogenesis and uptake of EVs. Exos are the smallest vesicles (30–150 nm in diameter) originating by endocytosis. MVs (also called microparticles) have size of 100–800 nm in diameter and are released from cell membrane by budding. ApoEVs (0.2–5 µm in diameter) are released from cell membrane surface in late stage of apoptosis. Membrane of Exos and MVs beside others contain MHC, tetraspanins CD9, CD63, CD81, and cell-specific receptor proteins. Exosomal cargo is enriched with broad range of RNAs, DNAs, and protein molecules

Since the first reference of EVs in platelet-free serum in 1946 by Chargaff et al*.* [4], the understanding of EVs biogenesis, structure, and functions has been significantly improved. According to the “Minimal information for studies of extracellular vesicles 2018” (MISEV2018), EVs is “the generic term for particles naturally released from the cell that are delimited by a lipid bilayer and cannot replicate, i.e., do not contain a functional nucleus” [5]. Historically EVs were classified to three major classes—Exos, microvesicles (MVs), and apoptotic bodies (ApoEVs) according to their cellular origin (Fig. 1) [3]. Exosomes and MVs are both released by healthy cells, whereas ApoEVs resulted from the apoptotic process, when the cell's cytoskeleton breaks up and causes the membrane to bulge outward. Exos with a size of 30–150 nm are the smallest subpopulation of EVs; they are released upon fusion of multivesicular bodies with the plasma membrane and further exocytosis. Due to their endocytotic origin, Exos are commonly enriched in endosome-associated proteins such as Rab GTPases, SNAREs, Annexins, and Flotillin. Some of these proteins (e.g., Alix and Tsg101) are commonly used as exosome markers. Tetraspanins family of membrane proteins (CD9, CD63, and CD81) is also abundantly present in Exos and considered to be used as a markers [3, 6, 7]. MVs are shed from the plasma membrane by budding; they vary in size between 100 and 800 nm and are enriched in CD63, CD81, and Annexin V proteins. The biggest in size population of EV are ApoEV, ranging in size between 200 and 5000 nm and expressing Annexin V [3, 6, 7]. Despite the big number of study focused on ApoEVs’ characterization, there are still striking discrepancies in the literature in the characterization and isolation of ApoEVs [8]. Since there are no specific markers for different subtypes of EVs, which would distinguish endosome-origin Exos and plasma membrane-derived MVs, International Society of EVs urged to consider use of operational terms for EV subtypes, which refer to physical characteristics of EVs (f.ex. size), or biochemical composition (f.ex. CD63^+^/CD81^+^-EVs) or cellular origin (f.ex. platelet EVs), unless authors can establish reliable specific markers of subcellular origin [5].

EVs have gained widespread interest due to their ability to carry bioactive components such as RNAs, DNA, and proteins. However, besides their luminal cargo, EVs can also carry a significant surface cargo encompassing DNA and especially proteins such as for example CD41^+^ for platelets, CD144^+^ for endothelial cells, or CD45^+^ for leukocytes derived EVs [9]. Both, Exos and MVs, are known to facilitate intercellular communication processes between cells in close proximity as well as distant cells. EVs cargo is actively loaded prior to the release from parental cell [6, 10, 11] and could significantly influence target cells' metabolism, function, and life span [6, 12, 13]. EVs can contain proteins such as cytokines, chemokines, heat shock and major histocompatibility complex (MHC) proteins, lipids, messenger RNA (mRNA), and microRNAs (miRNAs). Since EVs are present in most biological fluids (blood, urine, saliva, semen, bronchoalveolar lavage, bile, ascitic fluid, breast milk, and cerebrospinal fluid (CSF) [14, 15]), they hold promise as a diagnostic tool. They can be isolated from the small amount of biological fluids and clinical samples and their cargo, which represents tissue-specific molecules with higher stability, can serve as disease-specific biomarkers. Furthermore, since their release and composition can be modulated by environmental factors, they can also serve as markers for disease status and treatment outcomes [14, 15].

## EVs isolation and characterization methods

Similarity in some of EVs subtype characteristics (overlapping size, biochemistry, surface markers) makes search of disease-specific EVs biomarker technically challenging [3, 5]. The broad range of isolation and characterization methods together with inconsistence in the EVs definition in modern scientific literature provide additional complexity to this search [5]. Among different methods used for EVs isolation, there are four major groups focused on the isolation of the smallest subtype-Exos: ultracentrifugation, ultrafiltration, affinity, and osmotic precipitation-based methods. The most widely used method for exosome isolation is *differential (ultra-) centrifugation* [16]. The separation of Exos from different samples with this method is based on serial and differentiated centrifugation with g-forces rising up to 100.000*g*. Although differential centrifugation (ultracentrifugation) is effective for the isolation of Exos, the technique is time-consuming, labor-intensive, and heavily instrument-dependent for both research laboratories and clinical settings alike [17, 18]. *Density gradient ultracentrifugation* is a modification of this technique aimed at increased purity of isolated Exos. In this method, the sample is added to an inert gradient medium for centrifugal sedimentation and particles are separated on the basis of their buoyant densities by density gradient ultracentrifugation using sucrose or iodixanol [19, 20]. While the purity and quality of Exos isolation is increased with this technique, low yield due to the multiple-step protocols is commonly observed. *Ultrafiltration* is often used as a purification technique after EV isolation for example by ultracentrifuge. Depending on the size of MVs, this method allows the separation of Exos from proteins and other macromolecules. Nevertheless, micro-/ultrafiltration is also applicable for exosome isolation [11, 21]. This method is a fast, simple technique which does not need any expensive equipment and can concentrate large sample volumes [22–25]. However, it is characterized by lower exosome quality and suboptimal RNA purity as compared to ultracentrifugation [26]. Affinity-based *immunomagnetic beads isolation* method is based on the specific binding between monoclonal antibodies, loaded on magnetic beads and certain receptor molecules present on the surface of the Exos. Antibody coated beads against, for example, the tetraspanin proteins CD9, CD63, or CD81 are incubated with samples from which Exos are to be isolated. Then, the exosome–magnetic bead complexes are loaded onto a column, which is placed in a magnetic field. Therefore, the magnetically labeled Exos are retained within the column, while other cell components (unlabelled) run through [27]. The advantage of this approach is that it is target-specific and ensures the integrity of the extracted Exos. The method is also relatively easy to carry out and does not require expensive equipment. Additionally, this method allows selection and extraction of specific exosome fractions. However, the difficulties of exosome elution from the beads, and the need of sophisticated analytical tools to analyze Exos extracted from patient material together with expensive reagents make this method not user-friendly for point-of-care testing [20, 25, 27, 28]. *Osmotic precipitation—*an alternative technique that is increasingly being applied to isolate Exos—is the use of precipitants such as polyethylene glycol combined with low-speed centrifugation to pellet Exos for subsequent processing [29, 30]. This method is simple and fast and requires only a basic equipment [19, 20]; however, the purity of isolated exosome is compromised impairing downstream analysis. In addition, the polymer substance present in the isolate may interfere with downstream experiments [22, 31].

*Exosome characterization methods* could be divided into biophysical characterization of exosomal size range; molecular approaches to characterize the surface proteins and methods developed for analysis of exosomal cargo composition. The most common technique to determine the size and concentration of Exos in a sample is the *Nanoparticle Tracking Analysis (NTA).* The method is based on the Brownian motion of particles, which move rapidly in a liquid sample and act as point refractors when they pass through a laser beam. Videos can be recorded and a detailed differential particle-size distribution graph can be produced using analytical software [32]. A quite similar method is *Dynamic Light Scattering (DLS),* which is also based om the Brownian motion of small particles [27], but instead of scattered light, DLS uses the fluctuation in the intensity of scattered light to measure exosome size [33–35]. Another biophysical approach, commonly used to characterized morphology and size of the exosome is Electron microscopy [transmission (TEM) and scanning (SEM) electron microscopy] [36]. In addition, *Flow Cytometry* is often used to characterize EVs by mean of size, and absolute number; however, the limited sensitivity and resolution of flow cytometers should be considered. For smaller particles, such as Exos some approaches like the use of latex beads coated with monoclonal antibodies, which can bind and “pull-out” Exos can be introduced to allow their analysis by flow cytometry [37, 38]. Several methods have been developed for analysis of exosomal RNA cargo. Those methods include *microarray analysis, next-generation sequencing (NGS), and digital droplet PCR (ddPCR)* [36]*.* With regard to proteins, the protein content of Exos could be analyzed by *Western blotting, proteomic technology, and fluorescence-based cell sorting* [36]*.*

## The role of EVs during systemic inflammation and organ injury

Below, we provide an overview of the in vitro*, *in vivo and patients’ study, investigating the role of EVs as mediators, biomarkers, and/or therapeutics in traumatic injury. To avoid additional discrepancy in the nomenclature of EVs, we utilize the original author’s nomenclature used in the studies.

### EVs in sepsis/SIRS

According to the trauma register of the German Society of Traumatology (DGU-Polytraumaregister), more than 6% of multiple injured patients additionally develop septic complications and 20% of them develop multiple organ failure [39–41]. Sepsis is a systemic response of the immune system, in which Exos and MVs, originating from different type of cells, were shown to play diverse roles. Thus, the positive correlation of the increased production of platelet-MVs and poor outcome was shown in endotoxemia pig model [42]. In patients with septic shock, increased levels of platelet- and leukocyte-derived EVs and low level of endothelial cells-specific MVs were correlated with unfavorable outcome. One of the explanations of such correlation could be that in these patients, platelet-derived Exos are enriched with reactive oxygen species, which could induce vascular cell apoptosis [43]. Similarly, monocyte-derived microparticles (tissue factor+ and CD13^+^) were shown to be significantly increased in patients with trauma and severe sepsis [44]. This increase correlated significantly with the injury severity score (ISS) and acute physiology and chronic health evaluation score (APACHE II) in trauma patients; and with APACHE II and the international society of thrombosis and homeostasis (ISTH) overt disseminated intravascular coagulation (DIC) diagnostic criteria in sepsis patients [44]. In addition, MVs were shown to be important part of host protective mechanism in sepsis. Neutrophil-derived alpha-2-macroglobulin (A2MG)-containing MVs were shown to be elevated in plasma from patients with sepsis and their immunomodulatory role was verified in vivo. Administration of A2MG-enriched microparticles to mice with microbial sepsis provided protection against hypothermia, reduced bacterial titers, elevated immunoresolvent lipid mediator levels in inflammatory exudates, and reduced systemic inflammation [45].

Regardless of the role EVs are playing during the sepsis, it is less known, how different EVs could provide pro- or anti-inflammatory effects. According to the ExoCarta database (http://www.exocarta.org), Exos transfer cytokines, such as interleukins IL-1β, IL1α, IL-18, IL-32, IL-6, IL-8, macrophage migration inhibitory factor, tumor necrosis factor (TNF), vascular endothelial growth factor (VEGF), fractalkine, and chemokine ligands CCL2, CCL3, CCL4, CCL5, and CCL20; all known to be associated with the development of different inflammatory diseases [46]. Next to the inflammatory response via cytokines, also the complement system could play important role in EVs-associated effects [12]. It is well known that activation and interaction of the complement system with the other cascades such as coagulation lead to both pro- and anti-inflammatory reactions, which can affect morbidity and mortality in diseases, including trauma injury and sepsis [47–49]. MHC-bearing MVs could also function in this way; for example, it was described that non-survivors of septic shock exhibit increased numbers of EVs bearing complement component 5a receptor (C5aR) as compared with sepsis survivors [49].

In addition, EVs cargo miRNAs were shown to play an important role in mediation of sepsis in several in vitro*, *in vivo and patients’ studies. Recently, it was proposed and confirmed in sepsis mouse model that miRNAs from sepsis plasma Exos promote inflammation by inducing cytokine production via TLR7-MyD88 signaling [50]. The pro-inflammatory role of exosomal miR-155 was shown in LPS-induced sepsis in mice [51]. This was confirmed in in vitro study, where the treatment of RAW cells with miRNA-155 inhibitor results in significant reduction of LPS-induced TNFα production [52]. Also in septic patients, it was shown that miR-155 is associated with a high sepsis-related organ failure assessment (SOFA) score and correlates with the appearance of immunomodulating CD39^+^Tregs [53]. miR-146a was shown to play an opposite role and reduce the pro-inflammatory response in LPS-induced sepsis in mice [51].

Another view on possible role of EVs during the sepsis could be gained from the studies using EVs as therapeutics. It was shown that overexpressed in MSC-derived EVs, miR-223 mediates cardio-protection during sepsis via downregulation of Semaphorin-3A and Stat3 [54]. Pre-treatment of MSC with Il-1β was shown to enhance the production of miR-146a-enriched Exos, which leads to increase of septic mice survival [55]. LPS pre-treated MSC-derived Exos were suggested to have improved regulatory abilities for macrophage polarization and resolution of chronic inflammation by shuttling let-7b miRNA [56].

Summarizing the above, EVs play an important role in the development of sepsis and septic organ failure. In particular, EVs microRNAs miR-155 and miR-146a are mediators of inflammation, and might be good targets for future therapeutics, targeted on decrease of septic organ damage and mortality.

## EVs in injury and trauma

### EVs in traumatic brain injury

Traumatic brain injury (TBI) causes 37% of injury-related death in trauma patients [57]. Therefore, TBI is a major cause of mortality and morbidity particular in the younger population and is associated mainly with long-term-disabilities in survivors [58–60]. The cell–cell communication in the brain strongly depends on paracrine mechanisms mediated, in particular, via EVs [61], which are known to be released from all brain cells: neurons, astrocytes, microglia, and oligodendrocytes [62–65].

Neuroinflammation is an important step in TBI development, which was shown to be mediated by EVs [63]. For example, microglia-originated EVs, containing pro-inflammatory molecules, such as miR-155 and IL-1ß, are systemically detectable 24 h after TBI [63]. The role of EVs as a regulator of the immunological response in TBI was proofed in in vivo* experiments,* showing that transfer of astrocyte-shed EVs from inflammatory brain damaged animal into healthy animal leads to neuroinflammation [63]. An alteration in the permeability of the blood brain barrier is another crucial step in the development of TBI, in which EVs and their cargo could play a role. In case of systemic inflammation, TNF-α increase could lead to increased permeability of the blood–brain barrier that in turn make Exos able to cross the blood–brain barrier and therefore induce inflammatory processes in brain tissue [66]. miR-132-containing neuron-derived Exos were shown to regulate blood–brain barrier permeability by affecting expression of vascular endothelial cadherin [67]. EVs and EVs miRNAs were found to play role in blood–brain communication during peripheral inflammation. It was shown that choroid plexus epithelium cells could sense and transmit information about the peripheral inflammatory status to the central nervous system via the release of EVs into the cerebrospinal fluid, which transfer this pro-inflammatory message (miR-1a, miR-146, miR-9, and miR-155) to recipient brain cells [55].

Exosomal miRNAs play an important role in TBI establishment and usually have specific expression pattern during TBI that make them good candidates as biomarkers of this type of trauma. For example, it was shown that IL-1β-induced acute neuroinflammation and oxidative stress are characterized by the presence of astrocytes-released Exos. These Exos are enriched with specific subset of 22 miRNAs, known to influence inflammation and apoptosis via targeting BCL-1, TLR-4, BCL2L1, Bcl-2-associated X protein (BAX), and caspase 3 proteins [68]. In mice TBI model, the significant difference in the expression of miR-129-5p, miR-212-5p, miR-9-5p (all up-regulated in TBI) and miR-152-5p, miR-21, miR-374b-5p, and miR-664-3p (all down-regulated in TBI) was detected [69]. Moreover, the expression of miR-21 was shown to be increased at different time-points after TBI systemically and in neurons [69, 70]. In rodent TBI model, specific EVs miRNA expression pattern (significantly increased miR-21, miR-146, miR-7a, and miR-7b; decreased miR-212) indicates the presence of an enhancement loop among neuroinflammation and EVs [71]. In another study, around 50 exosomal miRNAs were described to be altered after TBI (31 up-regulated and 19 down-regulated) [72].

Beside miRNAs, also EVs cargo proteins might be considered as TBI biomarkers. The presence in exosomal cargo of classical neurotrauma biomarkers including ubiquitin C-terminal hydrolase L1 (UCHL1), Tau, Occludin, and amyloid β proteins was shown to be associated with a poor outcome and neurological deficit after TBI [73–75]. Exosomal Tau protein level was shown to correlate with cognitive, affective, and somatic post-concussive symptoms in US veterans with TBI [74]. Also exosomal IL-10 level correlates with behavioral symptoms after TBI [76]. In patients with severe TBI, MVs were shown to be enriched with glial fibrillary acid protein (GFAP) and aquarporin-4 [77], but not with neuron-specific enolase (NSE), although systemic increase of NSE is well known as a common biomarker of brain injury [77]. Generally, EV-derived markers of neuro-damage hold a great potential as TBI biomarkers as they provide more dynamic view of damage as common systemic biomarkers [76, 78].

MSC-derived exosome is considered as a cell-free therapeutic tool to reduce inflammatory consequences of TBI. Bone marrow stromal cells (BMSCs)-derived Exos were shown to inhibit expression of the pro-apoptotic protein BAX and the pro-inflammatory cytokines TNF-α and IL-1β, while enhancing the expression of the anti-apoptotic protein BCL-2, and thus to ameliorate early inflammatory response following brain injury [79]. Furthermore, BMSCs-Exos could modulate microglia/macrophage polarization by downregulating the expression of inducible nitric oxide synthase (INOS) and upregulating the expression of CD206 and arginase-1 [79]. MSC-derived Exos loaded with neuroprotective miR-216a-5p were shown to inhibit neuroinflammation and promote neuronal regeneration and in particular recovery of sensorimotor function and spatial learning ability [80]. MSC exosome-treated TBI rats show significant improvement in spatial learning as measured by the modified Morris water maze test and sensorimotor functional recovery [81]. Furthermore, exosome treatment was demonstrated to significantly increase the number of newly generated endothelial cells in the lesion boundary zone and dentate gyrus, as well as reduce neuroinflammation, promote angiogenesis, and reduce the damaging response after TBI [81]. Next to influencing neuroinflammation, EVs, released from hypoxia-pre-treated MSCs, could shift M1 to M2 phenotype in microglia. This phenotype switch, described as anti-inflammatory and neuroprotective, is mediated by EVs miR-216a-5p which inhibits TLR-4/NF-kB and activates phosphoinositid-3 kinase (PI3K)/Akt signaling pathways [82].

Summarizing the above, EVs could influence neuroinflammation, permeability of blood–brain barrier, autophagy, and polarization of microglia phenotype, and are therefore important players in TBI. EVs and EV cargo components hold great potential to be a diagnostic and therapeutic tool in TBI. However, until now, the most of the studies were conducted in animal models; therefore, the roles of EVs in TBI first need to be investigated in patients.

### EVs in acute cardiac injury

The incidence of cardiac contusion in patients with chest trauma ranges from 3 up to 76% [83, 84]. The definition of cardiac damage includes the increase of cardiac damage marker troponin and a functional impairment [85]. The traumatic damage of the heart is an independent predictor of a poor outcome including in-hospitalization, ICU-time, need for catecholamines, and death [86, 87]. Many cell types are known to be involved in a physiological heart function including cardiomyocytes, endothelial cells, fibroblasts, vascular smooth muscle cells, neuronal cells, immune cells, and stem cells [88]. The communication between these specific cells besides others is mediated via MVs.

Cardiomyocyte-shed EVs have a size ranging from 40 to 300 nm, and contain Caveolin-3 and Flotillin-1 proteins on the surface [89]. The cargo of these vesicles was shown to be enriched with sarcomeric and mitochondrial proteins, such as tropomyosin, myomesin, as well as cardiac-type myosin binding proteins [90]. The cargo and number of cardiomyocytes-released EVs could vary significantly as a reaction to the external stimulus and stress conditions, such as hypoxia, inflammation, or injury. Hypoxia was shown to be responsible for the increased release of TNF-α-containing Exos from cardiomyocytes [91]. These cardiomyocyte-derived EVs also contain heat shock protein HSP-60, which could be responsible for cardiomyocytes apoptosis and, via TLR activation, for pro-inflammatory reaction [88, 90]. Next to hypoxia, stimulation of cardiomyocytes with growth factors, such as transforming growth factor (TGF)-β, could lead to the enrichment of EVs cargo with TGF-signaling pathway proteins, which could mediate cellular hypertrophy and proliferation [92].

Beside the injury-mediating role, cardiomyocyte-shedded EVs could play protective and/or regenerative role. EV-derived miR-34a was shown to abolish the doxorubicin-induced cardiomyocyte senescence via upregulation of the phosphatase 1 nuclear targeting subunits [93]. EVs, derived from embryonic stem cell, are able to inhibit doxorubicin-induced cardiotoxicity by attenuation of TLR4-NLRP3 inflammasome-mediated cell death [94]. Cardiomyocyte-specific microRNAs are known to play an important role in the cardiac cell–cell communication [95]. miR-208 and miR-499, regulating the expression of sarcomeric genes, and miR-1 and miR-132, involved in ion channel regulation, were shown to have anti-apoptotic, anti-fibrotic, and anti-oxidative effects [96–101]. Exosomal miR-194 was found to impair mitochondrial activity in obese mice and cardiac function and reduced ejection fraction and increased N-terminal prohormone of brain natriuretic peptide (NT-proBNP) in humans [102].

EVs and their cargo might serve as markers of cardiac damage. In the experimental setting, it was shown that doxorubicin treatment of cardiomyocytes induce release of EVs, containing brain/heart isoforms of glycogen phosphate (PYGB). These specific EVs were detectable earlier as, for example, the common marker of cardiac injury-troponin and therefore suggested to be an early indicator of cardiac injury [103]. Several proteins were found to be up-regulated in EVs in plasma of myocardial infarction patients (complement C1qA, complement C5, apolipoprotein D, apolipoprotein C-III, platelet glycoprotein IB alpha chain, and platelet basic protein) [104]. These EVs are now discussed as potential new diagnostic tool in acute damage of the heart, for example, in myocardial infarction or traumatic cardiac injury [104].

Different authors consider MSC-derived EVs as a prominent therapeutic tool, because they were found to reduce apoptosis, increase cell proliferation, improve functional recovery, reduce infarct size and fibrosis, stimulate vascularization, and suppress inflammation after acute myocardial infarction [105]. The intramyocardial injection of MSC-EVs was shown to markedly enhance blood flow recovery, following by infarct size reduction and cardiac systolic and diastolic performance preservation in an acute myocardial infarction (MI) rat model [106]. In similar model, EVs derived from protein kinase B (Akt)-overexpressing MSC were found to provide significant pro-angiogenetic effect due to cargo of platelet‐derived growth factor D (PDGF‐D), known to strongly improve cardiac function [107]. By the regulation of Bcl-2 protein family expression human umbilical cord MSC-Exos were able to protect myocardial cells from apoptosis, promote angiogenesis, and, therefore, improve cardiac systolic function in acute myocardial infarction model [108]. In vitro, MSC-derived EVs were shown to stimulate cardiomyocytes proliferation and inhibition of H_2_O_2_-induced apoptosis [109]. The role of miRNAs in therapeutic effects of MSC-Exos was described in several studies. Thus, exosomal miR-19a was shown to target phosphatase and tensin homolog (PTEN) gene expression, which results in the activation of the Akt and ERK cell survival signaling pathways [110]. The anti-apoptotic role of exosomal miR-122 was described in the context of ischemic heart disease in vitro and in vivo [111].

Overall, cardiac EVs and their cargo play multiple roles during cardiac damage. They were shown to participate at different steps following injury that makes them good cardiac damage-biomarker candidates. Despite numerous studies focused on therapeutic effect of Exos in myocardial infarction, the study investigating the role of EVs/EVs cargo in traumatic cardiac contusion is scarce and needs further expansion.

### EVs in acute respiratory distress syndrome (ARDS)

ARDS is a life-threatening heterogeneous syndrome with complex pathology and mechanism. According to the Berlin Definition, ARDS is classified into mild, moderate, and severe stages, based on respiratory insufficiency, hypoxic respiratory failure, the Horovitz index, and bilateral pulmonary infiltrates detected radiographically [112]. 8.1% of intensive-care unit (ICU) patients develop an ARDS within the first 48 h after admission and therefore need an invasive mechanical ventilation [113]. The detailed knowledge about the mechanisms, mediators, and biomarkers of this disease will help clinicians to improve the diagnostics and treatment approaches for ARDS. The following section aims to summarize studies describing the roles of EVs in the ARDS development.

EVs in lung injury can originate from multiple cell types, including alveolar and bronchial epithelial cells, endothelial cells, alveolar macrophages, neutrophils, lymphocytes, fibroblasts, and blood cells [47]. The cell origin of EVs influence their cargo and therefore the role they are playing in the development of lung injury. The increased permeability of lung epithelium is one of the most important steps in the development of ARDS, likely triggered by endothelial cell-released EVs. In murine acute lung injury (ALI) model and in patients with severe sepsis-induced ALI, it was shown that MVs, originating from endothelial cells, are enriched with Sphingosine-1-phosphate receptor 3 (S1P3) protein, known to increase permeability of lung epithelium [114]. Similar EVs were detected in ventilation induced lung injury model and in endotoxin (LPS) exposure experiments in vitro which makes these EVs a good ALI biomarker candidate linked to disease severity and outcome [115]. The inverse correlation between elevated level of circulating EVs and the risk of ARDS development was found in septic cohort of critically ill patients [116]. Next to the endothelium, also other cell types are involved in EVs mediation of lung injury reaction. For example, neutrophil activation, playing a crucial role in the development of ARDS could be induced by EVs. It was shown that MVs derived from stored red blood cell induce neutrophils priming and activation, when injected in healthy mice, which further could lead to adverse effects, including lung injury [117]. In human, RBC-released EVs also lead to increase of CD11b expression in neutrophils, increased superoxide production, and enhanced phagocytotic ability [117]. Also, monocytes and macrophages were found to release EVs in the context of ARDS development. Monocyte-released EVs were shown to upregulate the synthesis of pro-inflammatory mediators via activation of NF-kB [118]. In the context of chest trauma, Shi et al. (2020) recently described that M1 macrophages, but not M0-derived EVs induce macrophage polarization [119].

The mechanism of EV-mediated ARDS is manifold and not fully investigated yet; nevertheless, some studies suggest that microRNAs could play crucial role in the development of acute lung injury. For example, miR-17/221 in shuttling EVs were shown to modulate macrophage integrin β1 recycling, which leads to macrophage recruitment and lung inflammation [120]. Also EVs miR-211 and miR-320 activate alveolar macrophages and initiate pro-inflammatory cytokine secretion [120]. Reduction of miR-425 in Exos of ARDS patients was found to causes aberrant fibroblast proliferation, collagen synthesis, and fibrosis via modulating TGF-β/Smad signaling [121]. EV-derived miR-126, miR-27a, miR-146a, and miR-155 were found to predict ARDS in patients with community-acquired pneumonia [122]. The presence of miR-92a-3p, miR-320a, miR-221-3p, miR-145p, miR-342-3p, miR-10-5p, and miR-422a in EVs was found to be specific for oxidative stress-induced ARDS [123]. In acid inhalation-induced lung injury model, miR-221-3p, miR-320a, miR-92a-3p, miR-17-5p and miR18-5p were enriched in EVs in bronchoalveolar lavage fluid (BALF) [120].

Next to the studies reporting lung injury-mediating role of EVs, there are few showing regenerative potential of EVs in the context of this injury. In LPS-induced ARDS model, MSCs derived EVs (CD44^+^) were shown to suppress cytokine production (TNF-alpha and IL-8), increase the number of M2 alveolar macrophages, and augment phagocytic ability of monocyte-derived macrophages [124]. Alveolar macrophages, pre-treated with MSC-EVs, and injected in LPS-treated mice were shown to reduce inflammation and lung injury [124].

### EVs in acute kidney injury

Acute kidney injury (AKI) is recognized as one of the most serious complications among hospitalized patients with acute illness and those undergoing major surgery [125]. According to the KDIGO (Kidney Disease Improving Global Outcomes) criteria, AKI is present in 18% of all hospitalized patients leading to in-hospital mortality of 11% [126]. EVs gained a wide attention as a source of pathogenic molecules, biomarkers, and therapeutic compounds in AKI. Within the kidney, EVs can originate from blood cells, podocytes or tubular epithelial cells, renal tubular cells, renal tissues, and glomerular endothelial cells, and could be detected within the circulation, urine, or in the kidney tissue (reviewed in [127]).

In several in vivo studies, urinary exosomal proteins and RNAs were found to be a potential biomarker for AKI diagnosis and severity prediction. For example, urinary exosomal AQP-1 and AQP-2 proteins and mRNAs were shown to be significantly decreased in animals with ischemia/reperfusion-induced AKI [128, 129], and aquaporins were found to reflect the progressive development of AKI [130]. miR-16, miR-24, and miR-200c in urinary Exos were correlated with an early (injury) phase, whereas miR-125 and miR-351 were found at the late (fibrotic) stage of AKI in ischemia/reperfusion injury rat model [131]. In patients’ study, EVs were suggested as potential marker of renal impairment. Thus, in patients with sepsis-induced AKI, an increase in total and platelet-derived (CD41^+^/CD13^+^) microparticles was reported [132]. Endothelial and leucocyte-derived MVs were found to be increased in patients with disseminated intravascular coagulation [133]. In septic patients, early systemic increase of vesicles was associated with improved survival [134]. Multiple organ failure during sepsis was associated with lower amount of platelet-derived EVs and high systemic concentrations of granulocyte- and erythrocyte-derived EVs [135].

In patients with kidney injury EVs, protein cargo is changed and could provide specific signature of this injury. EVs from patients with acute tubular necrosis have high levels of EV-derived Na^+^/H^+^-exchanger isoform 3 protein compared to healthy controls-EVs [136]. Fetuin A in urinary Exos of ICU patients was found to correlate with the appearance of AKI. Noteworthy, exosomal Fetuin A, but not free Fetuin A was detectable in the urine before morphologic injury developed [137]. The EV-derived activating transcription factor 3 (ATF3) was identified as marker of acute tubular injury [138]. In another study, Exos derived from patients with sepsis-induced AKI were shown to carry high amounts of neutrophil gelatinase-associated lipocalin (NGAL) and activating transcription factor 3 [139].

Another component of EVs cargo, microRNAs, was also found to be associated with renal function in kidney diseases. In urine from patients with chronic kidney disease, EV-derived microRNAs miR-29 and miR-200 were significantly reduced as compared to healthy individuals. This reduction was found to correlate with decreased renal function and the degree of tubular-intestinal fibrosis [140]. End-stage chronic kidney disease was found to be associated with overexpression of EV-derived miR-133, which is linked to inflammation and renal endothelial dysfunction [141]. Development of fibrosis in kidney diseases was shown to be associated with the microRNAs Let-7cp, miR-532-3p, miR-429, miR-143-3p, miR-770-5p, miR-224-5p, and let-7a-5p in plasma-derived Exos [142]. In patients with diabetic type I-caused kidney disease, miR-130a and miR-145a were significantly increased in urine Exos [143]. These observations show some correlation between a risk factor for the kidney disease comorbidities and exosomal microRNAs profile, but in case of AKI further studies needed to validate similar possibility.

There is an increasing evidence implicating the kidney-protective role of Exos, particularly those derived from MSCs, for attenuation and/or prevention of AKI. EVs from glomerular MSC were found to stimulate tubular regeneration in AKI [144]. EVs derived from human bone-marrow MSCs improved the recovery from AKI in a mouse model of glycerol-induced acute tubular injury and stimulate proliferation of tubular epithelial cells in vitro [145]. Furthermore, application of human bone-marrow- or umbical-cord MSC-Exos enhanced the recovery of renal function in a gentamicin or cisplatin-induced AKI models, and the protective effects were mediated by RNA carried by the Exos/MVs [146–148]. The critical role of microRNAs in exosome-promoted recovery after AKI was proofed in experiments via depletion of microRNAs in EVs in Drosha-knockdown cells [149].

To summarize, EVs not only play a role in the pathogenic mechanisms of kidney diseases, but also serves as the valuable source of potential non-invasive biomarkers for diagnostics and prognostics. Nevertheless, whereas some microRNAs were described as potential markers in chronic kidney disease, it is still little known about specific exosomal microRNA cargo in context of AKI. The MSC-released EVs provide the therapeutic potential in regeneration after AKI. It should be noted that there is still no study investigating EVs in trauma-induced AKI or rhabdomyolysis.

### EVs in liver injury

Liver is one of the most frequently injured organs in abdominal trauma. There is a paradigm shift in the management of liver trauma due to advancements of diagnostic and therapeutic modalities. Nowadays, the traditional standard biomarkers for liver injury are based on the measurement of hepatic enzymes in plasma or serum including AST, ALT, alkaline phosphatase (AP), and gamma-glutamil-transpeptidase (γGT) [150]. However, serum or plasma levels of these enzymes do not always reflect the stage of liver disease, therefore causing significant limitations in the diagnosis and staging of different chronic and acute liver disorders.

Both types of liver epithelia (i.e., hepatocytes and cholangiocytes), natural killer T (NKT) cells, hepatic stellate cells, Kupffer cells, adult liver stem cells, and hepatic sinusoidal endothelial cells are exosome-shedding and/or exosome-target cells [151–156]. In one proteomic analysis, it was shown that primary hepatocytes secrete exosome-like vesicles containing among others the proteins, specific only for hepatocyte-derived populations, such as ASGR receptor, apolipoproteins, and paraoxonases [157]. Moratti et al. demonstrated that plasma exosome sPTPRG protein represents a novel candidate protein biomarker whose increased expression is associated to hepatocyte damage [158]. Such specific molecular signature of liver-derived Exos may be useful to discriminate and purify hepatocyte-derived Exos from different body fluids to diagnose specific liver disease (non-alcoholic steatohepatitis, alcohol-related liver disease, cirrhosis, and hepatitis C virus infection) [3].

EVs miRNAs could also serve as a biomarker of liver injuries or chronic diseases. For example, increased serum levels of EVs miR-122 was shown to correlate with alanine aminotransferase (ALT) in different liver injury models (alcohol, paracetamol, and TLR9 ligands as CpG dinucleotid and LPS-induced) [7, 159]. miR-122, Let7f, and miR-34a were found in alcoholic steaotohepatitic liver-released EVs [160]. In patients with chronic liver disease, miR-122 and miR-192 appeared in systemic EVs, whereas a corresponding decrease in expression of these miRNAs was observed in the liver [161]. In drug-induced liver injury, stressed hepatocytes were shown to release miR-122 containing hepatocyte-derived Exos, which mediate an early immune response, also in the absence of overt hepatocellular toxicity [162]. Even subtoxic levels of acetaminophen were found to lead to significant changes in liver-specific RNAs in hepatocyte-derived EVs [162]. In alcohol-intoxicated trauma patients with a liver injury, the total number of EVs, EVs miR-122 and let7f, and several inflammatory cytokines like IL-6 and IL-33 were increased [163]. Furthermore, it was hypothesized and demonstrated in alcoholic steatohepatitis (ASH) mice model that hepatocyte-shedded EVs could contain unique miRNAs pattern, providing a specific “barcode” for the specific type of liver disease [3]. The pro-regenerative role of hepatocyte-derived Exos was shown in ischemia/reperfusion injury- and partial hepatectomy-mice models [164]. miRNA-214 in Exos, released from hepatic stellate cells, was found to be responsible for the regulation of CCN-2-dependent fibrogenesis [165]. EVs were found to play important role in the development of alcoholic liver disease (ALD) by increasing the numbers of inflammatory/M1 Kupffer cells and infiltrating monocytes while reducing the percentage of CD206^+^ CD163^+^ (anti-inflammatory/M2) Kupffer cells [166].

Intravenously injected Exos preferentially accumulate in the liver and reduce renal clearance, making them particularly suitable for the treatment of liver diseases [167]. In the case of liver injury, Exos liver uptake is enhanced [168], so that Exos can be rapidly and predominantly distributed in the liver to maximize their therapeutic effect. Studies have shown that Exos derived from different origin-MSCs inhibit the activation of related signaling pathways in acute liver failure (ALF). For example, human endometrial mesenchymal cell-derived Exos significantly reversed mouse ALF by up‐regulating STAT3 and inhibiting the NF‐κB pathway [169]. Exos isolated from adipose tissue-derived mesenchymal stem cells were found to significantly improve liver biochemical indicators in LPS/GalN‐induced fulminant hepatitis [170]. Human bone-marrow mesenchymal stem cell Exos attenuate hepatocyte apoptosis by promoting autophagy and, therefore, effectively reduce liver cell damage after ALF [171]. Chorionic plate‐derived MSCs Exos, which overexpress microRNA‐125b, help liver regeneration by inhibiting activation of Hedgehog (Hh) signaling [172].

In summary, due to specific molecular signature, liver-originated EVs are prominent novel biomarkers and therapeutic targets in different liver comorbidities.

### EVs in polytrauma

Trauma, often referred as society-neglected disease, is still one of the leading causes of death and disability worldwide, especially in the younger patients. With rising injury count and severity, mortality and morbidity arise, as well [173, 174]. Circulating in plasma EVs are of interests to physicians treating trauma patients as they could display an organ damage. They could be also involved in post-traumatic complications such as venous thromboembolism and promote development of infectious complications and multi-organ dysfunction syndrome. EVs also seems to play a crucial role in fracture healing in multi trauma patients, promoting cross talk in the process of coagulation, inflammation, angiogenesis, and osteogenesis (reviewed in [175]).

In one of the first study focused on the role of EVs in multiple trauma patients performed in 2001, Ogura et al*.* has shown that the amount of platelet-derived (CD62P^+^) microparticles was significantly increased in the sera of severely injured patients as compared to the healthy volunteers [176]. Authors confirmed the role of platelets-derived microparticles by showing that ionomycin increases microparticle formation and platelet-polymorphonuclear leukocyte- and monocytes-binding, whereas *N*-formyl-methionyl-leucyl-phenylalanine (FMLP), which affects the leukocytes, has no effect on the above parameters. The same group of authors has later demonstrated an increase of polymorphonuclear leukocyte (PMNL)-derived (CD11b^+^) microparticles in a comparable cohort of trauma patients as early as 2 days after trauma [177]. Trauma-induced coagulopathy following severe injury is known to be associated with increased bleeding and mortality. It was hypothesized that injury might result in alteration of cellular phenotypes and release of cell-derived microparticles, which have procoagulant, thrombin generation, and clotting functions. To better understand the body’s early response to trauma at a cellular level, Prospective, Observational, Multicenter, Major Trauma Transfusion (PROMMTT) study was aimed to characterize MP phenotypes and thrombin generation in severely injured trauma patients at admission, and relate significant changes to coagulopathy and bleeding [178]. The authors have found that while differences in platelet-derived microparticles counts did not reach statistical significance, red blood cell-derived, leukocyte-derived, and endothelial-derived microparticles counts were significantly higher in trauma patients compared to controls. Furthermore, in this study, the increased number of tissue factor-bearing MPs (TFMP) was found to be a predictor of substantial bleeding early after acute trauma [178]. In similar study, circulating procoagulant MVs of red cell (CD 235a/Annexin V^+^) and platelets (CD41^+^/Annexin V^+^) origin, rich in phospholipid, were found to be significantly elevated following traumatic injury and remained elevated at 72 h post injury. In contrast to other studies, endothelial cell- and leucocyte/tissue factor-MVs numbers were not elevated following trauma in these patients. Patients who died in this study due to pro-thrombotic disorder were found to have significantly reduced amount of procoagulant platelet-derived and red blood cell-derived MVs, that suggest the association between fewer CD41^+^/AnnV^+^ MVs, hypocoagulability, and mortality [48]. In 2017, Kuravi et al*.* provided insights in the total EVs load in blood of patients suffering severe traumatic injury, with follow-up from within hours up to 28 days [9]. The authors showed a significant increase in total number of circulating EVs and higher number of EVs derived from platelets (CD41^+^), leukocytes (CD45^+^) and endothelial cells (CD144^+^) in these trauma patients over the 28 days. Correlation between EVs count and clinical outcome of trauma patients was established by Matusmoto et al*.* [44]. They showed that an increased number of monocyte-derived TF^+^/CD13^+^ microparticles correlate significantly with ISS and APACHEII patients score in the acute phase in trauma patients which suggest that this type of MVs is important in the pathogenesis of early SIRS following trauma.

EVs of MSCs and platelets origin are currently considered as a potential treatment approach for trauma patients. In a mice model of hemorrhagic shock and lung injury, the systemic application of MSCs or MSC-derived EVs was shown to modulate cytoskeletal signaling and to attenuate lung vascular permeability after hemorrhagic shock [179]. Also platelet-derived EVs were found to decrease endothelial cell permeability and to restore endothelial cell junctions after thrombin challenge in vitro and in a tail snip hemorrhage model [180]. Results of the study in a rat model of traumatic hemorrhagic shock suggest that platelet-derived EVs could be a potential tool to improve the severe trauma outcome, as they maintain hemodynamic stability and attenuate uncontrolled bleeding [181].

Although some research showed participation of EVs in the development of multiple trauma consequences, none of them are focused on the characterizing the active component of these particles—cargo proteins and RNAs. These underlies the need for more detailed research in this field.

## EVs as diagnostic/prognostic markers in acute organ injury

**Fig. 2:**
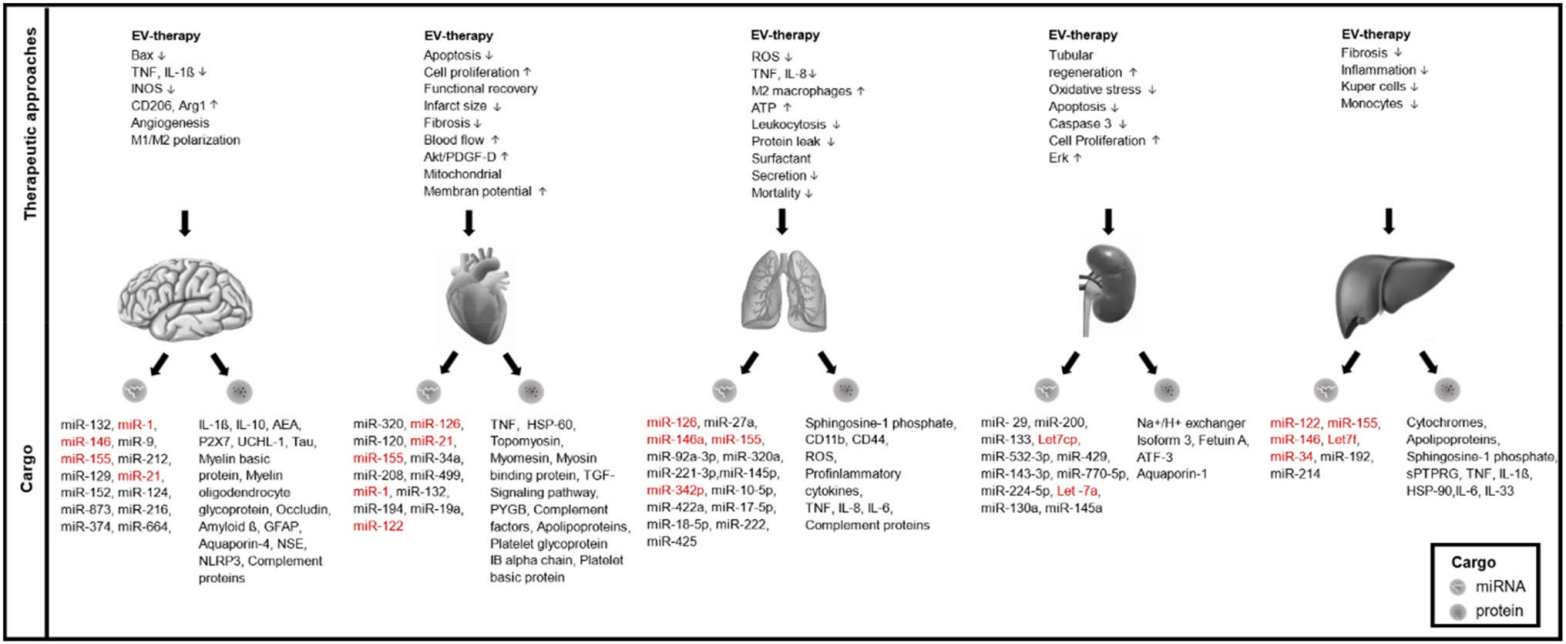
Extracellular vesicles in acute organ injuries: cargo content and therapeutic effects. Summary of the proteins and miRNAs cargo content of EVs in traumatic brain, acute cardiac, acute lung, acute kidney, and liver injuries. microRNAs which expressions were found to be most altered in organ trauma are highlighted in red. Up-regulated (arrow up) and down-regulated (arrow down) genes after EVs therapeutic treatments

Summarizing the above, the field of trauma/acute organ injury research is rapidly evolving; however, to date, there are no biomarkers that are associated with diverse aetiologies, clinical presentations, and degrees of severity of trauma injuries, which would help in treatment selection. Recent years showed a marked increase in the number of publications focused on the role of EVs as mediators and biomarkers of traumatic injuries. A good traumatic injury biomarker should be a specific, sensitive, and stable molecule, which can be obtained in a relatively non-invasive way and used to detect identity and severity of organ injury and predict prognosis. One good candidate for such marker are EVs miRNAs. For a long time due to their high abundance and their role as regulators of gene expression, circulating microRNAs have been proposed as potential markers in a broad spectrum of diseases [182]. EVs miRNAs, in comparison to circulating free miRNAs (often released from damaged/dying cells [183]), are released for intercellular communication, which is more stable due to encapsulation in lipid membrane and considered to be better liquid biopsy biomarkers for disease diagnosis and prognosis as circulating free miRNAs. In Table 1, we summarize the studies showing the presence of specific EVs miRNAs expression pattern in different traumatic injuries. In Fig. 2, we show that some of such miRNAs are specific for only one type of organ injury, whereas others (like miR-155, miR-126, miR-146, miR-21, and miR-122) could be found in EVs cargo in different organ injuries (red). Future study should be aimed to expand the list of such miRNAs as well as to proof their specificity and sensitivity as markers of individual organ injuries and polytrauma.

**Table 1:** Summary of EVs—microRNA (miRNAs) found in systemic inflammation and organ injury in in vitro*, *in vivo and patients studies

Disease	EV-derived miRNA	Role
*SIRS/sepsis*		
LPS-induced sepsis (mice) In vitro	miR-155 (pro-inflammatory miRNA)	Promotes endotoxin-induced inflammation in mice [51] Increases IL-6 production; increases TNF-α production and decreases SOCS1 mRNA expression in RAW macrophages stimulated with LPS in vitro [52]
LPS-induced sepsis (mice) CLP-induced sepsis	miR-146 (anti-inflammatory miRNA)	Reduces the pro-inflammatory response to LPS [51] miR-146a was transferred to macrophages, resulted in M2 polarization, and finally led to increased survival in septic mice [55]
Septic cardiomyopathy (in vitro)	miR-233 (anti-inflammatory)	miR-233 leads to downregulation of Sema3A and Stat3 leading to cardio protection (reduced inflammation/cell death) [54]
Treatment of LPS-induced sepsis	Let-7b (anti-inflammatory)	LPS‑preconditioned mesenchymal stromal cells modify macrophage polarization by exosomal Let-7b via TLR4/NF-κB/STAT3/AKT regulatory signaling pathway [56]
*TBI*		
Blood brain barrier permeability	miR-132	Influences adherent junction (VE-Cadherin) of the blood brain barrier, increases permeability [66, 67]
Systemic inflammation	miR-146, miR-155 (neuroinflammatory)	Systemic inflammation leads to increased release of EVs in cerebrospinal fluid containing neuroinflammatory miRNAs miR-146 and miR-155 [184]
TBI (rats)	miR-129-5p, miR-212–5p, miR-9-5p	Up-regulated after TBI [69, 72]
TBI (rats)	miR-152-5p, miR-21, miR-374b-5p	Down-regulated after TBI [69, 72]
TBI (mice)	miR-155 (neuroinflammatory)	Microglial-derived, contribute to progressive neuroinflammatory response [185]
TBI (mice)	miR-124-3p (anti-neuroinflammatory)	Inhibits neuronal inflammation; promote neurit outgrowth after scratch injury, improves neurologic outcome [186]
TBI (patients, mice, in vitro)	miR-873	Inhibits neuroinflammation by inhibition of NF-kB signaling and s transform M1 microglia into M2 phenotype [187]
Traumatic spinal cord injury	miR-216a-5p (MSC-derived, therapeutic)	Inhibits neuroinflammation, promotes neuronal regeneration, inhibits TLR-4/NF-kB, activates PI3K/Akt signaling pathway, initiates the shift of microglia M1 to M2 phenotype [82]
*Cardiac injury*		
Cardiac hypertrophy (in vitro)	miR-21	Downregulates sorbin and SH3 domain containing protein 2 cardiac hypertrophy [188]
Cardiac injury (mice)	miR-155 (inflammatory)	miR-155 inhibits fibroblast proliferation by downregulation of son of sevenless 1 expression, promotes inflammation [189]
Doxorubicin-induced cardiomyopathies (in vitro)	miR-34a	Abolishes the doxorubicin-induced cardiomyocyte senescence via upregulation of the phosphatase 1 nucelar targeting subunits [93]
Cardiac injury (obese mice)	miR-194	Impairs ATP production and basal oxygen consumption, impaired cardiac function, increased NT-proBNP in humans [102]
Hypoxic stress (in vitro)	miR-19a (anti-apoptotic)	Targets PTEN which results in the activation of the Akt and ERK cell survival signaling pathway reduces apoptosis [110]
Ischemic heart disease (mice)	miR-122 (anti-apoptotic, MSC-derived)	Anti-apoptotic by direct targeting of methyl CpG binding protein 2 [111]
*ARDS*		
Community-acquired pneumonia (patients)	miR-126, miR-27a, miR-146a and miR-155	Good predictors of ARDS development, miR-126 is a predictor of 28-days mortality [122]
Oxidative stress-induced ARDS (mice)	miR-320a, miR-221-3p, miR-145p, miR-342-3p	Up-regulated in oxidative-stress-induced ARDS [123]
Acid-induced ARDS (mice)	miR-221-3p, miR-17-5p (inflammatory)	miR-17/221 modulates macrophage ß1 integrin recycling macrophage recruitment lung inflammation [120]
ARDS	miR-211 and miR-320 (inflammatory)	Activate alveolar macrophages pro-inflammatory cytokines [120]
LPS lung macrophages (in vitro/mice)	miR-221/222	Epithelial cell proliferation [190]
*Acute kidney injury*		
Chronic kidney injury (patients)	miR-29, miR-200 (urine)	Correlates with renal function and the degree of tubular-intestinal fibrosis [140]
End-stage chronic kidney disease (patients)	miR-133 (plasma)	miRNA is linked to inflammation and renal endothelial dysfunction [141]
Kidney fibrosis (rats)	Let-7cp, miR-532-3p, miR-429 and let-7a-5p (plasma)	Up-regulated [142]
Diabetic kidney disease (diabetic patients)	miR-130a and miR-145a (urine)	Up-regulated [143]
*Acute liver injury*		
Liver injury (mice)	miR-122	Correlates with ALT in different liver injury models most abundant liver micro RNA [7, 159, 160]
Inflammatory liver disease (mice)	miR-155 and miR-146	Up-regulated in inflammatory liver diseases [159]
Alcoholic steaotohepatitis (patients + mice)	miR-122, Let7f, miR-34a	Up-regulated in plasma [160]
Chronic liver disease (mice)	miR-122 and miR-192	Up-regulation accompanied with decrease in the liver, promotes liver fibrosis in mice [161]
Liver injury (patients + mice)	miR-214	CCN-2-dependant fibrogenesis [165]
Drug-induced liver injury (rats)	miR-122	Early immune response, also in absence of overt hepatocellular toxicity [162]
Alcohol intoxication, liver injury (patients)	miR-122/let7f	Up-regulated together with the inflammatory cytokines IL-6 and IL-33 [163]

Other good candidates for such marker are cell-specific EVs in circulation, as they are bearing origin-specific subsets of proteins that likely correlated to cell-type-associated functions. EVs were shown to have protein signatures that closely reflect the associated clinical pathophysiology in cancer diseases [191], and it seems to be consistent to expect EVs with similar properties in other diseases and injuries. In case such EVs with specific protein signatures would be identified, they can add to the list of potential good biomarkers. We summarized cell-type specific EVs, detected in circulation in patients with systemic inflammation and with different organ injuries in Table 2. Further studies should enrich this list, proof their specificity, and describe their function.

**Table 2:** Cell-type specific EVs found in systemic inflammation and organ injury in in vitro*, *in vivo and patients studies

EVs -cell origin	Found in	Function	References
*SEPSIS/SIRS*			
Platelets	Endotoxemia pig model	Increase correlates with pure outcome	[42]
Monocytes (TP^+^/CD13^+^)	Patients with trauma or septic shock	Increase in trauma and sepsis correlates with ISS, APACHE II, and DIC criteria	[44]
Neutrophils (A2MG)	Patients with sepsis	Increased	[45]
Neutrophils (A2MG)	CLP in mice	Protect against hypothermia, reduce bacterial titers, elevate immunoresolvent lipid mediator levels in inflammatory exudates and reduced systemic inflammation	[45]
*TBI*			
Microglia	TBI (24 h after injury)	Neuroinflammation (Il-1β)	[63]
Microglia	Repetitive TBI in mice	Containing miR-124-3p which improve neurologic outcome and inhibit neuroinflammation	[186]
Astrocytes	TBI in mice	Neuroinflammation	[63]
Astrocytes	Patients with TBI and mouse model	Contain miR-873, transform M1 microglia into M2 phenotype	[187]
Astrocytes	Acute mild traumatic brain injury in patients	Astrocyte-derived EVs contain 12–35 fold higher levels of neurotoxic complement proteins compared to neuron-derived Exos	[192]
Choroid plexus epithelial cells	In vitro LPS treatment of cells	Pro-inflammatory reaction	[184]
*Cardiac injury*			
Cardiomyocytes	In vitro hypoxia	Contain HSP-60 leading to cardiomyocytes apoptosis and a pro-inflammatory reaction	[88, 90]
Cardiomyocytes	In vitro hypoxia	Contain TGF-signaling pathway proteins, which mediate cellular hypertrophy and proliferation	[92]
Cardiomyocytes	Doxorubicin-induced cardiomyocyte	Reduce cardiomyocyte senescence	[94]
Cardiomyocytes	In vitro ischemia*/*myocardial ischemia in rats/myocardial fibrosis in rats	Anti-apoptotic, anti-fibrotic and anti-oxidative effects	[96–101]
*ARDS*			
Endothelial cells	ALI in mice and ARDS in humans	Increase lung epitelium permeability	[114]
Red blood cells	Injected in healthy mice	Neutrophil activation (increased superoxide production and enhanced phagocytotic ability)	[117]
Monocytes/macrophages	In vitro: human lung epithelial cells	Synthesis of pro-inflammatory mediators	[118]
*AKI*			
Platelets (CD41^+^/CD13^+^)	Sepsis-induced AKI	Increased	[132]
Endothelial cells and leukocytes	DIC in patients	Disseminated intravascular coagulation	[133]
Platelets, granulocytes, erythrocytes	Patients with multiple organ failure	Decrease Increase Increase in multiple organ failure/SEPSIS	[135]
*Liver*			
Hepatocytes (ASGR receptor, apolipoproteins, paraoxonases and others specific proteins)	Basic proteomic studies	Contain ASGR receptor, apolipoproteins, paraoxonases and others specific proteins	[157]
Liver epithelia (i.e., hepatocytes and cholangiocytes), natural killer T (NKT) cells, hepatic stellate cells, Kupffer cells, adult liver stem cells, and hepatic sinusoidal endothelial cells	In vitro, liver toxicity, in hepatic fibrosis, cirrhosis, hepatocellular carcinoma	Are exosome-releasing and/or exosome-targeting cells	[151–156]
Hepatocytes	Drug-induced liver injury	Contain miR-122, mediate an early immune response also in absence of overt hepatocellular toxicity	[162]
Hepatocytes	Ischemia/reperfusion injury and partial hepatectomy-mice models	Mediate liver repair and regeneration	[164]
Hepatic stellate cells	fibrotic or steatotic livers, or in culture-activated or ethanol-treated primary mouse cells	Fibrogenesis	[165]
*Polytrauma*			
Platelets (CD62P^+^)	Serum of severe injured patients (mean ISS 33)	Increased in severe injured patients compared to healthy controls	[176]
PNML (CD11b^+^)	Trauma patients (mean ISS 30)	Systemically increased	[177]
Red blood (CD235a^+^), leukocytes (CD45^+^), endothelial cells (CD51^+^/CD144^+^)	Trauma patients (Mean ISS 26)	Increased significantly after trauma Tissue factor-bearing MPs (TFMP) was found to be a predictor of substantial bleeding early after acute trauma	[178]
Red cells (CD235a^+^/annv^+^) Platelets (CD41^+^/annv^+^)	Traumatic injury compared to controls after 72 h	Increased; fewer CD41^+^/AnnV^+^ MVs correlate with hypocoagulability and mortality	[48]
Platelets (CD41^+^) Leukocytes (CD45^+^) Endothelial cells (CD144^+^)	Trauma patients, SIRS	Correlation between EVs number and clinical outcome of trauma patients	[44]
Monocytes (TF^+^/CD13^+^)	Trauma patients, SIRS	Correlate with ISS; APACHEII, development of SIRS	[44]

## Conclusion

EVs with their unique miRNAs and proteins signatures are of great interest as biomarkers for the wide range of diseases and pathologies. We believe that scientific efforts in this field should be focused on development of simple and robust methods for isolation and characterization of circulating EVs; compilation and replenishment of databases, containing information about disease/injury-specific EVs, and such study should use standard and ubiquitous EVs nomenclature. If such combined efforts are made, we will soon receive a set of new biomarkers that will help accurately assess the level of trauma, predict the clinical outcome, and optimize the therapy for individual patients.

## References

[1] Vos T, Lim SS, Abbafati C, Abbas KM, Abbasi M, Abbasifard M, Abbasi-Kangevari M, Abbastabar H, Abd-Allah F, Abdelalim A, Abdollahi M (2020). Global burden of 369 diseases and injuries in 204 countries and territories, 1990–2019: a systematic analysis for the Global Burden of Disease Study 2019. Lancet (Lond, Engl).

[2] Xu W, Song Y (2017). Biomarkers for patients with trauma associated acute respiratory distress syndrome. Mil Med Res.

[3] Eguchi A, Kostallari E, Feldstein AE, Shah VH (2019). Extracellular vesicles, the liquid biopsy of the future. J Hepatol.

[4] Chargaff E, West R (1946). The biological significance of the thromboplastic protein of blood. J Biol Chem.

[5] Théry C, Witwer KW, Aikawa E, Alcaraz MJ, Anderson JD (2018). Minimal information for studies of extracellular vesicles 2018 (MISEV2018): a position statement of the International Society for Extracellular Vesicles and update of the MISEV2014 guidelines. J Extracell Vesicles.

[6] Lässer C, Jang SC, Lötvall J (2018). Subpopulations of extracellular vesicles and their therapeutic potential. Mol Aspects Med.

[7] Hirsova P, Ibrahim SH, Verma VK, Morton LA, Shah VH (2016). Extracellular vesicles in liver pathobiology. Small particles with big impact. Hepatology.

[8] Caruso S, Poon IKH (2018). Apoptotic cell-derived extracellular vesicles: more than just debris. Front Immunol.

[9] Kuravi SJ, Yates CM, Foster M, Harrison P, Hazeldine J (2017). Changes in the pattern of plasma extracellular vesicles after severe trauma. PLoS ONE.

[10] Yáñez-Mó M, Siljander PR-M, Andreu Z, Zavec AB, Borràs FE (2015). Biological properties of extracellular vesicles and their physiological functions. J Extracell Vesicles.

[11] Crescitelli R, Lässer C, Szabó TG, Kittel A, Eldh M (2013). Distinct RNA profiles in subpopulations of extracellular vesicles: apoptotic bodies, microvesicles and exosomes. J Extracell Vesicles.

[12] Karasu E, Eisenhardt SU, Harant J, Huber-Lang M (2018). Extracellular vesicles. Packages sent with complement. Front Immunol.

[13] Kalluri R, LeBleu VS. The biology, function, and biomedical applications of exosomes. Science. 2020;367(6478). 10.1126/science.aau6977 PMC7717626 32029601

[14] Hu G, Drescher KM, Chen X-M (2012). Exosomal miRNAs: biological properties and therapeutic potential. Front Genet.

[15] Gonda DD, Akers JC, Kim R, Kalkanis SN, Hochberg FH (2013). Neuro-oncologic applications of exosomes, microvesicles, and other nano-sized extracellular particles. Neurosurgery.

[16] Jeppesen DK, Hvam ML, Primdahl-Bengtson B, Boysen AT, Whitehead B (2014). Comparative analysis of discrete exosome fractions obtained by differential centrifugation. J Extracell Vesicles.

[17] Yu L-L, Zhu J, Liu J-X, Jiang F, Ni W-K (2018). A comparison of traditional and novel methods for the separation of exosomes from human samples. Biomed Res Int.

[18] Livshits MA, Livshts MA, Khomyakova E, Evtushenko EG, Lazarev VN (2015). Isolation of exosomes by differential centrifugation: theoretical analysis of a commonly used protocol. Sci Rep.

[19] Zhang M, Jin K, Gao L, Zhang Z, Li F (2018). Methods and technologies for exosome isolation and characterization. Small Methods.

[20] Li P, Kaslan M, Lee SH, Yao J, Gao Z (2017). Progress in exosome isolation techniques. Theranostics.

[21] Witwer KW, Buzás EI, Bemis LT, Bora A, Lässer C (2013). Standardization of sample collection, isolation and analysis methods in extracellular vesicle research. J Extracell Vesicles.

[22] Lobb RJ, Becker M, Wen SW, Wong CSF, Wiegmans AP (2015). Optimized exosome isolation protocol for cell culture supernatant and human plasma. J Extracell Vesicles.

[23] Merchant ML, Powell DW, Wilkey DW, Cummins TD, Deegens JK (2010). Microfiltration isolation of human urinary exosomes for characterization by MS. Proteomics Clin Appl.

[24] Cheruvanky A, Zhou H, Pisitkun T, Kopp JB, Knepper MA (2007). Rapid isolation of urinary exosomal biomarkers using a nanomembrane ultrafiltration concentrator. Am J Physiol Renal Physiol.

[25] Reiner AT, Witwer KW, van Balkom BWM, de Beer J, Brodie C (2017). Concise review: developing best-practice models for the therapeutic use of extracellular vesicles. Stem Cells Transl Med.

[26] Alvarez ML, Khosroheidari M, Kanchi Ravi R, DiStefano JK (2012). Comparison of protein, microRNA, and mRNA yields using different methods of urinary exosome isolation for the discovery of kidney disease biomarkers. Kidney Int.

[27] Doyle LM, Wang MZ (2019). Overview of extracellular vesicles, their origin, composition, purpose, and methods for exosome isolation and analysis. Cells.

[28] Hong CS, Muller L, Boyiadzis M, Whiteside TL (2014). Isolation and characterization of CD34+ blast-derived exosomes in acute myeloid leukemia. PLoS ONE.

[29] Zeringer E, Barta T, Li M (2015). Vlassov AV (2015) Strategies for isolation of exosomes. Cold Spring Harbor Protoc.

[30] Rider MA, Hurwitz SN, Meckes DG (2016). ExtraPEG: a polyethylene glycol-based method for enrichment of extracellular vesicles. Sci Rep.

[31] van Deun J, Mestdagh P, Sormunen R, Cocquyt V, Vermaelen K (2014). The impact of disparate isolation methods for extracellular vesicles on downstream RNA profiling. J Extracell Vesicles.

[32] Dragovic RA, Gardiner C, Brooks AS, Tannetta DS, Ferguson DJP (2011). Sizing and phenotyping of cellular vesicles using Nanoparticle Tracking Analysis. Nanomed Nanotechnol Biol Med.

[33] Filipe V, Hawe A, Jiskoot W (2010). Critical evaluation of Nanoparticle Tracking Analysis (NTA) by NanoSight for the measurement of nanoparticles and protein aggregates. Pharm Res.

[34] Palmieri V, Lucchetti D, Gatto I, Maiorana A, Marcantoni M (2014). Dynamic light scattering for the characterization and counting of extracellular vesicles: a powerful noninvasive tool. J Nanopart Res.

[35] Frisken BJ (2001). Revisiting the method of cumulants for the analysis of dynamic light-scattering data. Appl Opt.

[36] Gurunathan S, Kang M-H, Jeyaraj M, Qasim M, Kim J-H (2019). Review of the isolation, characterization, biological function, and multifarious therapeutic approaches of exosomes. Cells.

[37] Ko J, Carpenter E, Issadore D (2016). Detection and isolation of circulating exosomes and microvesicles for cancer monitoring and diagnostics using micro-/nano-based devices. Analyst.

[38] Szatanek R, Baj-Krzyworzeka M, Zimoch J, Lekka M, Siedlar M (2017). The methods of choice for extracellular vesicles (EVs) characterization. Int J Mol Sci.

[39] Fleischmann C, Thomas-Rueddel DO, Hartmann M, Hartog CS, Welte T (2016). Hospital incidence and mortality rates of sepsis. Deutsches Arzteblatt Int.

[40] Moreira J (2013). Severe sepsis and septic shock. N Engl J Med.

[41] Huber-Lang M (2018). Sepsis nach polytrauma. Trauma Berufskrankh.

[42] Eriksson M, Nelson D, Nordgren A, Larsson A (1998). Increased platelet microvesicle formation is associated with mortality in a porcine model of endotoxemia. Acta Anaesthesiol Scand.

[43] Janiszewski M, Do Carmo AO, Pedro MA, Silva E, Knobel E (2004). Platelet-derived exosomes of septic individuals possess proapoptotic NAD(P)H oxidase activity. A novel vascular redox pathway. Crit Care Med.

[44] Matsumoto H, Yamakawa K, Ogura H, Koh T, Matsumoto N (2017). Clinical significance of tissue factor and CD13 double-positive microparticles in sirs patients with trauma and severe sepsis. Shock.

[45] Dalli J, Norling LV, Montero-Melendez T, Federici Canova D, Lashin H (2014). Microparticle alpha-2-macroglobulin enhances pro-resolving responses and promotes survival in sepsis. EMBO Mol Med.

[46] Keerthikumar S, Chisanga D, Ariyaratne D, Al Saffar H, Anand S (2016). ExoCarta. A web-based compendium of exosomal cargo. J Mol Biol.

[47] Lee H, Abston E, Zhang D, Rai A, Jin Y (2018). Extracellular vesicle. An emerging mediator of intercellular crosstalk in lung inflammation and injury. Front Immunol.

[48] Curry N, Raja A, Beavis J, Stanworth S, Harrison P (2014). Levels of procoagulant microvesicles are elevated after traumatic injury and platelet microvesicles are negatively correlated with mortality. J Extracell Vesicles.

[49] Unnewehr H, Rittirsch D, Sarma JV, Zetoune F, Flierl MA (2013). Changes and regulation of the C5a receptor on neutrophils during septic shock in humans. J Immunol (Baltimore, MD.:1950)..

[50] Xu J, Feng Y, Jeyaram A, Jay SM, Zou L (2018). Circulating plasma extracellular vesicles from septic mice induce inflammation via MicroRNA- and TLR7-dependent mechanisms. J Immunol (Baltimore, Md.:1950).

[51] Alexander M, Hu R, Runtsch MC, Kagele DA, Mosbruger TL (2015). Exosome-delivered microRNAs modulate the inflammatory response to endotoxin. Nat Commun.

[52] Momen-Heravi F, Bala S, Bukong T, Szabo G (2014). Exosome-mediated delivery of functionally active miRNA-155 inhibitor to macrophages. Nanomed Nanotechnol Biol Med.

[53] Liu J, Shi K, Chen M, Xu L, Hong J (2015). Elevated miR-155 expression induces immunosuppression via CD39(+) regulatory T-cells in sepsis patient. Int J Infect Dis.

[54] Wang X, Gu H, Qin D, Yang L, Huang W (2015). Exosomal miR-223 contributes to mesenchymal stem cell-elicited cardioprotection in polymicrobial sepsis. Sci Rep.

[55] Song Y, Dou H, Li X, Zhao X, Li Y (2017). Exosomal miR-146a contributes to the enhanced therapeutic efficacy of interleukin-1β-primed mesenchymal stem cells against sepsis. Stem Cells (Dayton, Ohio).

[56] Ti D, Hao H, Tong C, Liu J, Dong L (2015). LPS-preconditioned mesenchymal stromal cells modify macrophage polarization for resolution of chronic inflammation via exosome-shuttled let-7b. J Transl Med.

[57] Majdan M, Plancikova D, Brazinova A, Rusnak M, Nieboer D (2016). Epidemiology of traumatic brain injuries in Europe: a cross-sectional analysis. Lancet Public Health.

[58] Adekoya N, Thurman DJ, White DD, Webb KW (2002). Surveillance for traumatic brain injury deaths—United States, 1989–1998. Morb Mortal Wkly Rep Surveill Summ (Washington, D.C.:2002).

[59] Maas AIR, Stocchetti N, Bullock R (2008). Moderate and severe traumatic brain injury in adults. Lancet Neurol.

[60] Brooks JC, Strauss DJ, Shavelle RM, Paculdo DR, Hammond FM (2013). Long-term disability and survival in traumatic brain injury: results from the National Institute on Disability and Rehabilitation Research Model Systems. Arch Phys Med Rehabil.

[61] Panaro MA, Benameur T, Porro C (2020). Extracellular vesicles miRNA cargo for microglia polarization in traumatic brain injury. Biomolecules.

[62] Lachenal G, Pernet-Gallay K, Chivet M, Hemming FJ, Belly A (2011). Release of exosomes from differentiated neurons and its regulation by synaptic glutamatergic activity. Mol Cell Neurosci.

[63] Dickens AM, Tovar-Y-Romo LB, Yoo S-W, Trout AL, Bae M et al. Astrocyte-shed extracellular vesicles regulate the peripheral leukocyte response to inflammatory brain lesions. Sci Signaling. 2017;10(473). 10.1126/scisignal.aai7696 PMC5590230 28377412

[64] Hooper C, Sainz-Fuertes R, Lynham S, Hye A, Killick R (2012). Wnt3a induces exosome secretion from primary cultured rat microglia. BMC Neurosci.

[65] Frühbeis C, Fröhlich D, Kuo WP, Amphornrat J, Thilemann S (2013). Neurotransmitter-triggered transfer of exosomes mediates oligodendrocyte-neuron communication. PLoS Biol.

[66] Chen CC, Liu L, Ma F, Wong CW, Guo XE (2016). Elucidation of exosome migration across the blood-brain barrier model in vitro. Cell Mol Bioeng.

[67] Xu B, Zhang Y, Du X-F, Li J, Zi H-X (2017). Neurons secrete miR-132-containing exosomes to regulate brain vascular integrity. Cell Res.

[68] Gayen M, Bhomia M, Balakathiresan N, Knollmann-Ritschel B (2020). Exosomal MicroRNAs released by activated astrocytes as potential neuroinflammatory biomarkers. Int J Mol Sci.

[69] Ko J, Hemphill M, Yang Z, Sewell E, Na YJ (2018). Diagnosis of traumatic brain injury using miRNA signatures in nanomagnetically isolated brain-derived extracellular vesicles. Lab Chip.

[70] Lei P, Li Y, Chen X, Yang S, Zhang J (2009). Microarray based analysis of microRNA expression in rat cerebral cortex after traumatic brain injury. Brain Res.

[71] Harrison EB, Hochfelder CG, Lamberty BG, Meays BM, Morsey BM (2016). Traumatic brain injury increases levels of miR-21 in extracellular vesicles. Implications for neuroinflammation. FEBS Open Bio.

[72] Wang P, Ma H, Zhang Y, Zeng R, Yu J (2020). Plasma Exosome-derived MicroRNAs as novel biomarkers of traumatic brain injury in rats. Int J Med Sci.

[73] Gill J, Mustapic M, Diaz-Arrastia R, Lange R, Gulyani S (2018). Higher exosomal tau, amyloid-beta 42 and IL-10 are associated with mild TBIs and chronic symptoms in military personnel. Brain Inj.

[74] Kenney K, Qu B-X, Lai C, Devoto C, Motamedi V (2018). Higher exosomal phosphorylated tau and total tau among veterans with combat-related repetitive chronic mild traumatic brain injury. Brain Inj.

[75] Goetzl EJ, Elahi FM, Mustapic M, Kapogiannis D, Pryhoda M (2019). Altered levels of plasma neuron-derived exosomes and their cargo proteins characterize acute and chronic mild traumatic brain injury. FASEB J.

[76] Stern RA, Tripodis Y, Baugh CM, Fritts NG, Martin BM (2016). Preliminary study of plasma exosomal tau as a potential biomarker for chronic traumatic encephalopathy. J Alzheimer's Dis JAD.

[77] Nekludov M, Bellander B-M, Gryth D, Wallen H, Mobarrez F (2017). Brain-derived microparticles in patients with severe isolated TBI. Brain Inj.

[78] Peskind ER, Kraemer B, Zhang J (2015). Biofluid biomarkers of mild traumatic brain injury: whither plasma tau. JAMA Neurol.

[79] Ni H, Yang S, Siaw-Debrah F, Hu J, Wu K (2019). Exosomes derived from bone mesenchymal stem cells ameliorate early inflammatory responses following traumatic brain injury. Front Neurosci.

[80] Xu H, Jia Z, Ma K, Zhang J, Dai C (2020). Protective effect of BMSCs-derived exosomes mediated by BDNF on TBI via miR-216a-5p. Med Sci Monit.

[81] Zhang Y, Chopp M, Meng Y, Katakowski M, Xin H (2015). Effect of exosomes derived from multipluripotent mesenchymal stromal cells on functional recovery and neurovascular plasticity in rats after traumatic brain injury. J Neurosurg.

[82] Liu W, Rong Y, Wang J, Zhou Z, Ge X (2020). Exosome-shuttled miR-216a-5p from hypoxic preconditioned mesenchymal stem cells repair traumatic spinal cord injury by shifting microglial M1/M2 polarization. J Neuroinflamm.

[83] Sybrandy KC, Cramer MJM, Burgersdijk C (2003). Diagnosing cardiac contusion: old wisdom and new insights. Heart (British Cardiac Society).

[84] El-Chami MF, Nicholson W, Helmy T (2008). Blunt cardiac trauma. J Emerg Med.

[85] Kalbitz M, Schwarz S, Weber B, Bosch B, Pressmar J (2017). Cardiac depression in pigs after multiple trauma—characterization of posttraumatic structural and functional alterations. Sci Rep.

[86] Kalbitz M, Pressmar J, Stecher J, Weber B, Weiss M (2017). The role of troponin in blunt cardiac injury after multiple trauma in humans. World J Surg.

[87] Huber S, Biberthaler P, Delhey P, Trentzsch H, Winter H (2014). Predictors of poor outcomes after significant chest trauma in multiply injured patients: a retrospective analysis from the German Trauma Registry (Trauma Register DGU®). Scand J Trauma Resusc Emerg Med.

[88] Chistiakov DA, Orekhov AN, Bobryshev YV (2016). Cardiac extracellular vesicles in normal and infarcted heart. Int J Mol Sci.

[89] Waldenström A, Gennebäck N, Hellman U, Ronquist G (2012). Cardiomyocyte microvesicles contain DNA/RNA and convey biological messages to target cells. PLoS ONE.

[90] Malik ZA, Kott KS, Poe AJ, Kuo T, Chen L (2013). Cardiac myocyte exosomes: stability, HSP60, and proteomics. Am J Physiol Heart Circul Physiol.

[91] Yu X, Deng L, Wang D, Li N, Chen X (2012). Mechanism of TNF-α autocrine effects in hypoxic cardiomyocytes: initiated by hypoxia inducible factor 1α, presented by exosomes. J Mol Cell Cardiol.

[92] Gennebäck N, Hellman U, Malm L, Larsson G, Ronquist G (2013). Growth factor stimulation of cardiomyocytes induces changes in the transcriptional contents of secreted exosomes. J Extracel Vesicles.

[93] Liu Y, Liu Z, Xie Y, Zhao C, Xu J (2019). Serum extracellular vesicles retard H9C2 cell senescence by suppressing miR-34a expression. J Cardiovasc Transl Res.

[94] Tavakoli Dargani Z, Singla DK (2019). Embryonic stem cell-derived exosomes inhibit doxorubicin-induced TLR4-NLRP3-mediated cell death-pyroptosis. Am J Physiol Heart Circul Physiol.

[95] Li C, Pei F, Zhu X, Duan DD, Zeng C (2012). Circulating microRNAs as novel and sensitive biomarkers of acute myocardial Infarction. Clin Biochem.

[96] Xu C, Lu Y, Pan Z, Chu W, Luo X (2007). The muscle-specific microRNAs miR-1 and miR-133 produce opposing effects on apoptosis by targeting HSP60, HSP70 and caspase-9 in cardiomyocytes. J Cell Sci.

[97] He B, Xiao J, Ren A-J, Zhang Y-F, Zhang H (2011). Role of miR-1 and miR-133a in myocardial ischemic postconditioning. J Biomed Sci.

[98] Castoldi G, Di Gioia CRT, Bombardi C, Catalucci D, Corradi B (2012). MiR-133a regulates collagen 1A1: potential role of miR-133a in myocardial fibrosis in angiotensin II-dependent hypertension. J Cell Physiol.

[99] Li X, Wang J, Jia Z, Cui Q, Zhang C (2013). MiR-499 regulates cell proliferation and apoptosis during late-stage cardiac differentiation via Sox6 and cyclin D1. PLoS ONE.

[100] Izarra A, Moscoso I, Levent E, Cañón S, Cerrada I (2014). miR-133a enhances the protective capacity of cardiac progenitors cells after myocardial infarction. Stem Cell Rep.

[101] Li S, Xiao F-Y, Shan P-R, Su L, Chen D-L (2015). Overexpression of microRNA-133a inhibits ischemia-reperfusion-induced cardiomyocyte apoptosis by targeting DAPK2. J Hum Genet.

[102] Nie H, Pan Y, Zhou Y (2018). Exosomal microRNA-194 causes cardiac injury and mitochondrial dysfunction in obese mice. Biochem Biophys Res Commun.

[103] Yarana C, Carroll D, Chen J, Chaiswing L, Zhao Y (2018). Extracellular vesicles released by cardiomyocytes in a doxorubicin-induced cardiac injury mouse model contain protein biomarkers of early cardiac injury. Clin Cancer Res.

[104] Cheow ESH, Cheng WC, Lee CN, de Kleijn D, Sorokin V (2016). Plasma-derived extracellular vesicles contain predictive biomarkers and potential therapeutic targets for myocardial ischemic (MI) injury. Mol Cell Proteomics.

[105] Börger V, Bremer M, Ferrer-Tur R, Gockeln L, Stambouli O (2017). Mesenchymal stem/stromal cell-derived extracellular vesicles and their potential as novel immunomodulatory therapeutic agents. Int J Mol Sci.

[106] Bian S, Zhang L, Duan L, Wang X, Min Y (2014). Extracellular vesicles derived from human bone marrow mesenchymal stem cells promote angiogenesis in a rat myocardial infarction model. J Mol Med (Berlin, Germany).

[107] Ma J, Zhao Y, Sun L, Sun X, Zhao X (2017). Exosomes derived from Akt-modified human umbilical cord mesenchymal stem cells improve cardiac regeneration and promote angiogenesis via activating platelet-derived growth factor D. Stem Cells Transl Med.

[108] Zhao Y, Sun X, Cao W, Ma J, Sun L (2015). Exosomes derived from human umbilical cord mesenchymal stem cells relieve acute myocardial ischemic injury. Stem Cells Int.

[109] Shao L, Zhang Y, Lan B, Wang J, Zhang Z (2017). MiRNA-sequence indicates that mesenchymal stem cells and exosomes have similar mechanism to enhance cardiac repair. Biomed Res Int.

[110] Yu B, Kim HW, Gong M, Wang J, Millard RW (2015). Exosomes secreted from GATA-4 overexpressing mesenchymal stem cells serve as a reservoir of anti-apoptotic microRNAs for cardioprotection. Int J Cardiol.

[111] Feng Y, Huang W, Wani M, Yu X, Ashraf M (2014). Ischemic preconditioning potentiates the protective effect of stem cells through secretion of exosomes by targeting Mecp2 via miR-22. PLoS ONE.

[112] Eworuke E, Major JM, Gilbert McClain LI (2018). National incidence rates for Acute Respiratory Distress Syndrome (ARDS) and ARDS cause-specific factors in the United States (2006–2014). J Crit Care.

[113] Bellani G, Laffey JG, Pham T, Fan E, Brochard L (2016). Epidemiology, patterns of care, and mortality for patients with acute respiratory distress syndrome in intensive care units in 50 countries. JAMA.

[114] Sun X, Singleton PA, Letsiou E, Zhao J, Belvitch P (2012). Sphingosine-1-phosphate receptor-3 is a novel biomarker in acute lung injury. Am J Respir Cell Mol Biol.

[115] Letsiou E, Sammani S, Zhang W, Zhou T, Quijada H (2015). Pathologic mechanical stress and endotoxin exposure increases lung endothelial microparticle shedding. Am J Respir Cell Mol Biol.

[116] Shaver CM, Woods J, Clune JK, Grove BS, Wickersham NE (2017). Circulating microparticle levels are reduced in patients with ARDS. Crit Care (Lond, Engl).

[117] Belizaire RM, Prakash PS, Richter JR, Robinson BR, Edwards MJ (2012). Microparticles from stored red blood cells activate neutrophils and cause lung injury after hemorrhage and resuscitation. J Am Coll Surg.

[118] Neri T, Armani C, Pegoli A, Cordazzo C, Carmazzi Y (2011). Role of NF-kappaB and PPAR-gamma in lung inflammation induced by monocyte-derived microparticles. Eur Respir J.

[119] Shi Y, Luo P, Wang W, Horst K, Bläsius F (2020). M1 but not M0 extracellular vesicles induce polarization of RAW264.7 macrophages via the TLR4-NFκB pathway in vitro. Inflammation.

[120] Lee H, Zhang D, Wu J, Otterbein LE, Jin Y (2017). Lung epithelial cell-derived microvesicles regulate macrophage migration via MicroRNA-17/221-induced integrin β(1) recycling. J Immunol (Baltimore, Md.:1950).

[121] Wang L, Liu J, Xie W, Li G, Yao L (2019). miR-425 reduction causes aberrant proliferation and collagen synthesis through modulating TGF-β/Smad signaling in acute respiratory distress syndrome. Int J Clin Exp Pathol.

[122] Wu X, Wu C, Gu W, Ji H, Zhu L (2019). Serum exosomal MicroRNAs predict acute respiratory distress syndrome events in patients with severe community-acquired pneumonia. Biomed Res Int.

[123] Lee H, Zhang D, Zhu Z, Dela Cruz CS, Jin Y (2016). Epithelial cell-derived microvesicles activate macrophages and promote inflammation via microvesicle-containing microRNAs. Sci Rep.

[124] Morrison TJ, Jackson MV, Cunningham EK, Kissenpfennig A, McAuley DF (2017). Mesenchymal stromal cells modulate macrophages in clinically relevant lung injury models by extracellular vesicle mitochondrial transfer. Am J Respir Crit Care Med.

[125] Rewa O, Bagshaw SM (2014). Acute kidney injury-epidemiology, outcomes and economics. Nat Rev Nephrol.

[126] Zeng X, McMahon GM, Brunelli SM, Bates DW, Waikar SS (2014). Incidence, outcomes, and comparisons across definitions of AKI in hospitalized individuals. Clin J Am Soc Nephrol.

[127] Thongboonkerd V (2019). Roles for exosome in various kidney diseases and disorders. Front Pharmacol.

[128] Sonoda H, Yokota-Ikeda N, Oshikawa S, Kanno Y, Yoshinaga K (2009). Decreased abundance of urinary exosomal aquaporin-1 in renal ischemia-reperfusion injury. Am J Physiol Renal Physiol.

[129] Asvapromtada S, Sonoda H, Kinouchi M, Oshikawa S, Takahashi S (2018). Characterization of urinary exosomal release of aquaporin-1 and -2 after renal ischemia-reperfusion in rats. Am J Physiol Renal Physiol.

[130] Nielsen S, Frøkiaer J, Marples D, Kwon T-H, Agre P (2002). Aquaporins in the kidney: from molecules to medicine. Physiol Rev.

[131] Sonoda H, Lee BR, Park K-H, Nihalani D, Yoon J-H (2019). miRNA profiling of urinary exosomes to assess the progression of acute kidney injury. Sci Rep.

[132] Tőkés-Füzesi M, Woth G, Ernyey B, Vermes I, Mühl D (2013). Microparticles and acute renal dysfunction in septic patients. J Crit Care.

[133] Delabranche X, Boisramé-Helms J, Asfar P, Berger A, Mootien Y (2013). Microparticles are new biomarkers of septic shock-induced disseminated intravascular coagulopathy. Intensive Care Med.

[134] Soriano AO, Jy W, Chirinos JA, Valdivia MA, Velasquez HS (2005). Levels of endothelial and platelet microparticles and their interactions with leukocytes negatively correlate with organ dysfunction and predict mortality in severe sepsis. Crit Care Med.

[135] Joop K, Berckmans RJ, Nieuwland R, Berkhout J, Romijn FP (2001). Microparticles from patients with multiple organ dysfunction syndrome and sepsis support coagulation through multiple mechanisms. Thromb Haemost.

[136] Du Cheyron D, Daubin C, Poggioli J, Ramakers M, Houillier P (2003). Urinary measurement of Na+/H+ exchanger isoform 3 (NHE3) protein as new marker of tubule injury in critically ill patients with ARF. Am J Kidney Dis.

[137] Zhou H, Pisitkun T, Aponte A, Yuen PST, Hoffert JD (2006). Exosomal Fetuin-A identified by proteomics. A novel urinary biomarker for detecting acute kidney injury. Kidney Int.

[138] Chen H-H, Lai P-F, Lan Y-F, Cheng C-F, Zhong W-B (2014). Exosomal ATF3 RNA attenuates pro-inflammatory gene MCP-1 transcription in renal ischemia-reperfusion. J Cell Physiol.

[139] Panich T, Chancharoenthana W, Somparn P, Issara-Amphorn J, Hirankarn N (2017). Urinary exosomal activating transcriptional factor 3 as the early diagnostic biomarker for sepsis-induced acute kidney injury. BMC Nephrol.

[140] Lv L-L, Cao Y-H, Ni H-F, Xu M, Liu D (2013). MicroRNA-29c in urinary exosome/microvesicle as a biomarker of renal fibrosis. Am J Physiol Renal Physiol.

[141] Cavallari C, Dellepiane S, Fonsato V, Medica D, Marengo M (2019). Online hemodiafiltration inhibits inflammation-related endothelial dysfunction and vascular calcification of uremic patients modulating miR-223 expression in plasma extracellular vesicles. J Immunol (Baltimore, Md.:1950).

[142] Xie JX, Fan X, Drummond CA, Majumder R, Xie Y (2017). MicroRNA profiling in kidney disease Plasma versus plasma-derived exosomes. Gene.

[143] Barutta F, Tricarico M, Corbelli A, Annaratone L, Pinach S (2013). Urinary exosomal microRNAs in incipient diabetic nephropathy. PLoS ONE.

[144] Ranghino A, Bruno S, Bussolati B, Moggio A, Dimuccio V (2017). The effects of glomerular and tubular renal progenitors and derived extracellular vesicles on recovery from acute kidney injury. Stem Cell Res Ther.

[145] Bruno S, Grange C, Deregibus MC, Calogero RA, Saviozzi S (2009). Mesenchymal stem cell-derived microvesicles protect against acute tubular injury. J Am Soc Nephrol.

[146] Reis LA, Borges FT, Simões MJ, Borges AA, Sinigaglia-Coimbra R (2012). Bone marrow-derived mesenchymal stem cells repaired but did not prevent gentamicin-induced acute kidney injury through paracrine effects in rats. PLoS ONE.

[147] Zhou Y, Xu H, Xu W, Wang B, Wu H (2013). Exosomes released by human umbilical cord mesenchymal stem cells protect against cisplatin-induced renal oxidative stress and apoptosis in vivo and in vitro. Stem Cell Res Ther.

[148] Bruno S, Grange C, Collino F, Deregibus MC, Cantaluppi V (2012). Microvesicles derived from mesenchymal stem cells enhance survival in a lethal model of acute kidney injury. PLoS ONE.

[149] Collino F, Bruno S, Incarnato D, Dettori D, Neri F (2015). AKI Recovery induced by mesenchymal stromal cell-derived extracellular vesicles carrying MicroRNAs. J Am Soc Nephrol.

[150] Kim WR, Flamm SL, Di Bisceglie AM, Bodenheimer HC (2008). Serum activity of alanine aminotransferase (ALT) as an indicator of health and disease. Hepatology (Baltimore, MD).

[151] Royo F, Schlangen K, Palomo L, Gonzalez E, Conde-Vancells J (2013). Transcriptome of extracellular vesicles released by hepatocytes. PLoS ONE.

[152] Rodríguez-Suárez E, Gonzalez E, Hughes C, Conde-Vancells J, Rudella A (2014). Quantitative proteomic analysis of hepatocyte-secreted extracellular vesicles reveals candidate markers for liver toxicity. J Proteomics.

[153] Masyuk AI, Masyuk TV, LaRusso NF (2013). Exosomes in the pathogenesis, diagnostics and therapeutics of liver diseases. J Hepatol.

[154] Chen Y, Zeng Z, Shen X, Wu Z, Dong Y (2016). MicroRNA-146a-5p negatively regulates pro-inflammatory cytokine secretion and cell activation in lipopolysaccharide stimulated human hepatic stellate cells through inhibition of toll-like receptor 4 signaling pathways. Int J Mol Sci.

[155] Witek RP, Yang L, Liu R, Jung Y, Omenetti A (2009). Liver cell-derived microparticles activate hedgehog signaling and alter gene expression in hepatic endothelial cells. Gastroenterology.

[156] Fonsato V, Collino F, Herrera MB, Cavallari C, Deregibus MC (2012). Human liver stem cell-derived microvesicles inhibit hepatoma growth in SCID mice by delivering antitumor microRNAs. Stem Cells (Dayton, Ohio).

[157] Conde-Vancells J, Rodriguez-Suarez E, Embade N, Gil D, Matthiesen R (2008). Characterization and comprehensive proteome profiling of exosomes secreted by hepatocytes. J Proteome Res.

[158] Moratti E, Vezzalini M, Tomasello L, Giavarina D, Sorio C (2015). Identification of protein tyrosine phosphatase receptor gamma extracellular domain (sPTPRG) as a natural soluble protein in plasma. PLoS ONE.

[159] Bala S, Petrasek J, Mundkur S, Catalano D, Levin I (2012). Circulating microRNAs in exosomes indicate hepatocyte injury and inflammation in alcoholic, drug-induced, and inflammatory liver diseases. Hepatology (Baltimore, MD).

[160] Eguchi A, Lazaro RG, Wang J, Kim J, Povero D (2017). Extracellular vesicles released by hepatocytes from gastric infusion model of alcoholic liver disease contain a MicroRNA barcode that can be detected in blood. Hepatology (Baltimore, MD).

[161] Kostallari E, Hirsova P, Prasnicka A, Verma VK, Yaqoob U (2018). Hepatic stellate cell-derived platelet-derived growth factor receptor-alpha-enriched extracellular vesicles promote liver fibrosis in mice through SHP2. Hepatology (Baltimore, MD).

[162] Holman NS, Mosedale M, Wolf KK, LeCluyse EL, Watkins PB (2016). Subtoxic alterations in hepatocyte-derived exosomes an early step in drug-induced liver injury?. Toxicol Sci.

[163] Eguchi A, Franz N, Kobayashi Y, Iwasa M, Wagner N (2019). Circulating extracellular vesicles and their miR "Barcode" differentiate alcohol drinkers with liver injury and those without liver injury in severe trauma patients. Front Med.

[164] Nojima H, Freeman CM, Schuster RM, Japtok L, Kleuser B (2016). Hepatocyte exosomes mediate liver repair and regeneration via sphingosine-1-phosphate. J Hepatol.

[165] Chen L, Charrier A, Zhou Y, Chen R, Yu B (2014). Epigenetic regulation of connective tissue growth factor by MicroRNA-214 delivery in exosomes from mouse or human hepatic stellate cells. Hepatology (Baltimore, MD).

[166] Saha B, Momen-Heravi F, Furi I, Kodys K, Catalano D (2018). Extracellular vesicles from mice with alcoholic liver disease carry a distinct protein cargo and induce macrophage activation through heat shock protein 90. Hepatology (Baltimore, MD).

[167] Zhang Y-N, Poon W, Tavares AJ, McGilvray ID, Chan WCW (2016). Nanoparticle-liver interactions: cellular uptake and hepatobiliary elimination. J Control Release.

[168] Haga H, Yan IK, Takahashi K, Matsuda A, Patel T (2017). Extracellular vesicles from bone marrow-derived mesenchymal stem cells improve survival from lethal hepatic failure in mice. Stem Cells Transl Med.

[169] Tan CY, Lai RC, Wong W, Dan YY, Lim S-K (2014). Mesenchymal stem cell-derived exosomes promote hepatic regeneration in drug-induced liver injury models. Stem Cell Res Ther.

[170] Liu Y, Lou G, Li A, Zhang T, Qi J (2018). AMSC-derived exosomes alleviate lipopolysaccharide/d-galactosamine-induced acute liver failure by miR-17-mediated reduction of TXNIP/NLRP3 inflammasome activation in macrophages. EBioMedicine.

[171] Zhao S, Liu Y, Pu Z (2019). Bone marrow mesenchymal stem cell-derived exosomes attenuate D-GaIN/LPS-induced hepatocyte apoptosis by activating autophagy in vitro. Drug Des Dev Ther.

[172] Hyun J, Wang S, Kim J, Kim GJ, Jung Y (2015). MicroRNA125b-mediated Hedgehog signaling influences liver regeneration by chorionic plate-derived mesenchymal stem cells. Sci Rep.

[173] Fröhlich M, Lefering R, Probst C, Paffrath T, Schneider MM (2014). Epidemiology and risk factors of multiple-organ failure after multiple trauma: an analysis of 31,154 patients from the TraumaRegister DGU. J Trauma Acute Care Surg.

[174] Hildebrand F, Giannoudis PV, van Griensven M, Zelle B, Ulmer B (2005). Management of polytraumatized patients with associated blunt chest trauma: a comparison of two European countries. Injury.

[175] Qiao Z, Greven J, Horst K, Pfeifer R, Kobbe P (2018). Fracture healing and the underexposed role of extracellular vesicle-based cross talk. Shock (Augusta, Ga.).

[176] Ogura H, Kawasaki T, Tanaka H, Koh T, Tanaka R (2001). Activated platelets enhance microparticle formation and platelet-leukocyte interaction in severe trauma and sepsis. J Trauma.

[177] Fujimi S, Ogura H, Tanaka H, Koh T, Hosotsubo H (2003). Increased production of leukocyte microparticles with enhanced expression of adhesion molecules from activated polymorphonuclear leukocytes in severely injured patients. J Trauma.

[178] Matijevic N, Wang Y-WW, Wade CE, Holcomb JB, Cotton BA (2014). Cellular microparticle and thrombogram phenotypes in the Prospective Observational Multicenter Major Trauma Transfusion (PROMMTT) study: correlation with coagulopathy. Thromb Res.

[179] Potter DR, Miyazawa BY, Gibb SL, Deng X, Togaratti PP (2018). Mesenchymal stem cell-derived extracellular vesicles attenuate pulmonary vascular permeability and lung injury induced by hemorrhagic shock and trauma. J Trauma Acute Care Surg.

[180] Miyazawa B, Trivedi A, Togarrati PP, Potter D, Baimukanova G (2019). Regulation of endothelial cell permeability by platelet-derived extracellular vesicles. J Trauma Acute Care Surg.

[181] Lopez E, Srivastava AK, Burchfield J, Wang Y-W, Cardenas JC (2019). Platelet-derived- extracellular vesicles promote hemostasis and prevent the development of hemorrhagic shock. Sci Rep.

[182] Mori MA, Ludwig RG, Garcia-Martin R, Brandão BB, Kahn CR (2019). Extracellular miRNAs: from biomarkers to mediators of physiology and disease. Cell Metab.

[183] Arroyo JD, Chevillet JR, Kroh EM, Ruf IK, Pritchard CC (2011). Argonaute2 complexes carry a population of circulating microRNAs independent of vesicles in human plasma. Proc Natl Acad Sci USA.

[184] Balusu S, van Wonterghem E, de Rycke R, Raemdonck K, Stremersch S (2016). Identification of a novel mechanism of blood-brain communication during peripheral inflammation via choroid plexus-derived extracellular vesicles. EMBO Mol Med.

[185] Kumar A, Stoica BA, Loane DJ, Yang M, Abulwerdi G (2017). Microglial-derived microparticles mediate neuroinflammation after traumatic brain injury. J Neuroinflamm.

[186] Huang S, Ge X, Yu J, Han Z, Yin Z (2018). Increased miR-124-3p in microglial exosomes following traumatic brain injury inhibits neuronal inflammation and contributes to neurite outgrowth via their transfer into neurons. FASEB J.

[187] Long X, Yao X, Jiang Q, Yang Y, He X (2020). Astrocyte-derived exosomes enriched with miR-873a-5p inhibit neuroinflammation via microglia phenotype modulation after traumatic brain injury. J Neuroinflamm.

[188] Bang C, Batkai S, Dangwal S, Gupta SK, Foinquinos A (2014). Cardiac fibroblast-derived microRNA passenger strand-enriched exosomes mediate cardiomyocyte hypertrophy. J Clin Investig.

[189] Wang C, Zhang C, Liu L, A X, Chen B, (2017). Macrophage-derived mir-155-containing exosomes suppress fibroblast proliferation and promote fibroblast inflammation during cardiac injury. Mol Ther.

[190] Zhu Z, Zhang D, Lee H, Menon AA, Wu J (2017). Macrophage-derived apoptotic bodies promote the proliferation of the recipient cells via shuttling microRNA-221/222. J Leukoc Biol.

[191] Rontogianni S, Synadaki E, Li B, Liefaard MC, Lips EH (2019). Proteomic profiling of extracellular vesicles allows for human breast cancer subtyping. Commun Biol.

[192] Goetzl EJ, Yaffe K, Peltz CB, Ledreux A, Gorgens K (2020). Traumatic brain injury increases plasma astrocyte-derived exosome levels of neurotoxic complement proteins. FASEB J.

